# Plasma proteome profiling of cardiotoxicity in patients with diffuse large B-cell lymphoma

**DOI:** 10.1186/s40959-021-00092-0

**Published:** 2021-02-03

**Authors:** Charlott Mörth, Amal Abu Sabaa, Eva Freyhult, Christina Christersson, Jamileh Hashemi, Nashmil Hashemi, Masood Kamali-Moghaddam, Daniel Molin, Martin Höglund, Anna Eriksson, Gunilla Enblad

**Affiliations:** 1 Department of Immunology, Genetics & Pathology, Uppsala University, Rudbecklaboratoriet, 75185 Uppsala, Sweden; 2 Centre for Clinical Research Sörmland, Uppsala University, Uppsala, Sweden; 3 Department of Medical Sciences, National Bioinformatics Infrastructure Sweden, Science for Life Laboratory, Uppsala University, Uppsala, Sweden; 4 Department of Medical Sciences, Uppsala University, Uppsala, Sweden; 5 Department of Cardiology, Danderyd Hospital, Karolinska Institute, Stockholm, Sweden; 6 Department of Immunology, Genetics & Pathology, Science for Life Laboratory, Uppsala University, Uppsala, Sweden; 7 Center for Research and Development, Uppsala University/Region Gävleborg, Uppsala, Sweden

**Keywords:** Proteomics, Lymphoma, Cardiac toxicity, Doxorubicin

## Abstract

**Background:**

Cardiovascular toxicity is a notorious complication of doxorubicin (DXR) therapy for diffuse large B-cell lymphoma (DLBCL). Although surveillance of well-known biological markers for cardiovascular disease (CVD) as NTproBNP and Troponins may be helpful, there are no established markers to monitor for evolving CVD during treatment. New possibilities have arisen with the emergence of newer techniques allowing for analysis of plasma proteins that can be associated with cardiovascular disease. Proximity Extension Assay is one of them.

**Objectives:**

We aimed to illustrate the incidence of CVD in DLBCL patients treated with DXR and to establish whether there are plasma proteins associated with pre-existing or emerging CVD.

**Methods:**

In 95 patients, 182 different proteins from OLINK panels, NTproBNP, Troponin I and CRP were assessed prior to, during and after treatment. For comparison, samples from controls were analyzed.

**Results:**

In the DLBCL cohort, 33.3% had pre-treatment CVD compared to 5.0% in the controls and 23.2% developed new CVD. Of the 32.6% who died during follow up, CVD was the cause in 4 patients. Spondin-1 (SPON-1) correlated to pre-treatment CVD (1.22 fold change, 95% CI 1.10–1.35, *p* = 0.00025, q = 0.045). Interleukin-1 receptor type 1 (IL-1RT1) was associated to emerging CVD (1.24 fold change, 95% CI 1.10–1.39, *p* = 0.00044, q = 0.082).

**Conclusion:**

We observed a higher prevalence of CVD in DLBCL patients compared to controls prior to DXR therapy. Two proteins, SPON-1 and IL-1RT1, were related to pre-existing and emerging CVD in DXR treated patients. If confirmed in larger cohorts, IL-1RT1 may emerge as a reliable biomarker for unfolding CVD in DLBCL.

**Supplementary Information:**

The online version contains supplementary material available at 10.1186/s40959-021-00092-0.

## Introduction

The standard of care for diffuse large B-cell lymphoma (DLBCL) is CHOP (cyclophosphamide, doxorubicin (DXR), vincristine, and prednisone) combined with the anti-CD 20 monoclonal antibody; rituximab (R) [1–3]. The upholding of a high relative dose intensity (RDI) of CHOP has been associated with better progression-free survival (PFS) and overall survival (OS) in DLBCL [4–7].

The dose-limiting toxicity for DXR is early to late-onset heart damage manifesting as arrythmias, ischemia, systolic dysfunction and heart failure. The injury may present clinically weeks to decades after treatment. The individual tolerance to DXR differs and genetic factors, prior cardiac damage, tissue ischemia and other concomitant cardiac risk factors may affect the susceptibility to DXR damage [8–10]. Also rituximab alone has been associated to cardiotoxicity by causing exacerbations of angina, arrhythmias and heart failure [11, 12]. Furthermore, common risk factors for cardiovascular disease (CVD) among lymphoma patients, such as obesity, might increase the risk of heart failure even further [13].

There are limited ways to predict the specific risk for developing cardiotoxicity of each DLBCL patient treated with DXR in the clinical setting, and it is even harder to measure and address ongoing toxicity during treatment. Clinical risk assessment and heart ultrasound pre-treatment might help to select cases unsuitable for DXR.

In the last decade, circulating biomarkers, such as C reactive protein (CRP), N-terminal pro B-type natriuretic peptide (NT-proBNP) and Troponins have been introduced in screening for asymptomatic left ventricular (LV) dysfunction [14–16]. In DXR treated malignancies, some studies point at the possibility of using Troponin I as a predictor for heart failure, but find little evidence of the usefulness of NT-proBNP [17–20]. Contradictory to this, NT-proBNP improves the prediction of DXR induced cardiotoxicity when used in combination with a clinical risk score assessment function (FRESCO) in DLBCL [21].

New possibilities have arisen with the technology to analyze sets of proteins in serum or plasma samples. The proximity extension assay (PEA) is an immunoassay where large number of proteins can be measured simultaneously in small volumes of liquid tissues [22] (http://www.olink.com/), enabling the monitoring of patterns of proteins associated with cardiovascular disease (CVD) and cancer (ONC). Studies using this method have indicated its clinical utility in detecting new proteins associated with pre-existing or emerging heart failure as well as monitoring worsening of heart failure [23–27]. To our knowledge though, PEA has not yet been tested for the prediction of cardiac/cardiovascular toxicity before, during and after lymphoma treatment.

Our hypothesis was that new protein biomarkers for CVD could improve the assessment of cardiac and cardiovascular risk among DXR treated DLBCL. In the present exploratory study, we measured plasma levels of 182 circulating CVD- and tumor related (Olink CVD III and ONC II panel) proteins in a total of 186 samples (at baseline and follow-up) from 95 DLBCL patients treated with R-CHOP/R-CHOEP (CHOP plus etoposide). In addition, we analyzed the more established heart failure markers NTproBNP and Troponin I in 133 and CRP in 92 samples. For comparison samples from 60 non-DLBCL controls were obtained.

## Materials and methods

### Ethical approval

The U-CAN project, including this study, has been approved by the Regional Ethics Committee of Uppsala-Örebro (Ups 2012/198, 210/198/1, 2014/233). Data collection in the EpiHealth study and usage of the material in this project has been approved by the EC of Uppsala (Dnr 2010/402: 2010-12-01, 2011-11-17, 2015/179). The EpiHealth study is approved by the Swedish Data Protection Authority.

### Study design

The Uppsala-Umeå Comprehensive Cancer Consortium (U-CAN), is a high-quality longitudinal biobank with the sequential collection of clinical data as well as blood and tissue samples from cancer patients [28]. Patients aged ≥18 years and diagnosed with DLBCL between 2010 and 2015 were included in the U-CAN biobank at the time of diagnosis. Plasma samples for analysis of PEA of two OLINK™ multiplex protein panels (CVDIII and ONCII, for now on referred to as PEA-CO), as well as NTpro-BNP, and Troponin I were collected at diagnosis and for some patients during treatment and after completion of therapy and in follow-up. Plasma samples were also collected from age and gender-matched non DLBCL controls.

### Patient population

Ninety-five patients with a newly diagnosed DLBCL were included, all whom were planned for curative intent R-CHO(E)P therapy, although seven cases never received any DXR. Clinical information was obtained from the U-CAN database and from the patients’ medical records.

**Fig. 1:**
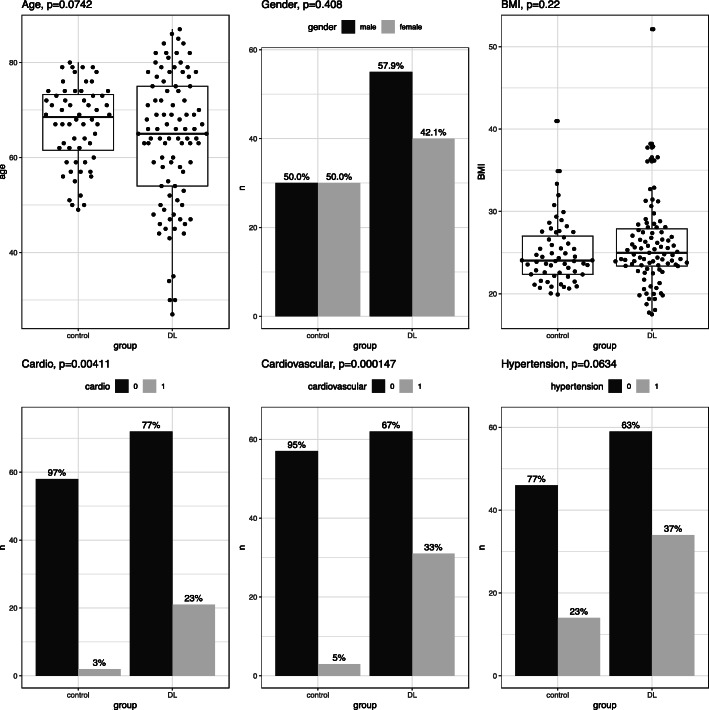
Characteristics of DLBCL patients vs controls

For comparison, plasma samples from 60 non DLBCL controls, 30 male and 30 non-pregnant females, age 49–80 years, median 67 years (Fig. 1), were obtained from the EpiHealth biobank. EpiHealth is an open-access, multicenter, longitudinal, cohort study investigating the interaction between genes and life-style factors possibly related to the development of common diseases in the adult population [29].

### Definitions

The data set included age at diagnosis, date of diagnosis, gender, international prognostic index (IPI) [30, 31], DXR dose per m^2^, smoking status (current, former, never), body mass index (BMI, weight in kg divided by square of height in meters), estimated glomerular filtration rate (eGFR, according to Lund-Malmö, mL/min/1.72m^2^) [32, 33] separated into levels 0–4 (eGFR> 90 = 0, eGFR60–89 = 1, eGFR30–59 = 2, eGFR15–29 = 3, eGFR< 15 = 4), CRP (normal/elevated according to the local laboratory), diabetes mellitus (DM), ongoing medications, date of relapse, date of death, cause of death (determined by information in charts or in death certificates). Patients dying during treatment or in relapse were coded as death due to lymphoma.

Cardiac disease was divided in the following categories; heart failure, angina pectoris, cardiac arrest, fibrillation, clinically relevant valvular disease, pacemaker and PCI treatment. Vascular disease was categorized as; claudicatio, carotid stenosis, aortic aneurysm surgery, deep vein thrombosis, pulmonary embolism, arterial thrombosis/ischemic stroke or cerebral hemorrhage, transient ischemic attack or surgery for aneurysm.

Cardiovascular disease (CVD) was defined as having a pre-existing or developing a diagnosis of any cardiac or vascular disease. Several patients had more than one diagnosis. An emerging new diagnosis was counted for regardless if the patient already had a CVD.

The occurrence of ongoing or emerging diagnoses were extracted from available information in the patients records. For one patient, the patient record lacked data on existence of CVD. For the control cohort, information regarding earlier or ongoing CVD and hypertension was available except for the categories deep vein thrombosis and pulmonary embolism.

For plasma samples, the date of collection divided the samples into samples taken before treatment initiation (pre-treatment), samples collected during ongoing treatment and samples after completion of therapy. Samples obtained at diagnosis before treatment initiation included 96 PEA-CO and 32 NTproBNP and Troponin I, during treatment 30 PEA-CO and 25 NTproBNP and Troponin I, at end of treatment 60 PEA-CO and 76 NTproBNP and Troponin I (Table 1). All but one patient had a sample taken before treatment. For patients with more than one pre-treatment sample, the earliest was used in the analysis of correlation to pre-existing or emerging cardiac disease or CVD. In 2 patients the first sample was taken the day after initiation of treatment and those were regarded as pre-treatment samples. For one patient, the exact date of start of treatment was unknown, the sample was excluded from the pre-treatment analysis. When evaluating protein level changes during treatment, the most recent sample before start of treatment and the first sample after completion of therapy was used in the case that several samples had been collected. Information regarding CRP at diagnosis was obtained from clinical records and available in 92 cases.

**Table 1:** Number of protein samples

	PEA-CO^a^	NTproBNP Troponin I^b^	CRP
Before	96^c^	32	92
During	30	25	0
After	60	76	0
Total	186	133	92

^a^ PEA-CO, proximity extension assay of 182 proteins. ^b^NTproBNP and Troponin I ^c^ 96 samples before start of treatment

from 94 patients. Two patients had two samples before and for one patient there was no sample and for one patient date of starting treatment was uncertain

### Multiplex proximity extension assays (PEA) and specimen characteristics

The PEA technology was utilized to assess plasma samples (1 μl) using the Olink™ multiplex protein panel Cardiovascular III (CVDIII) and Oncology II (ONCII).

Each panel consists of 92 human proteins where CVDIII includes cardiovascular- and inflammatory-related markers as well as some exploratory human proteins believed to be associated with cardiovascular disease. All proteins are further subclassified according to class, disease area and tissue expression based on public-access bioinformatic databases such as Uniprot and the Human Protein Atlas. The subset of proteins for each panel discussed in this paper are presented in Supplementary Table 1 and supplement 1 (CVDIII) and 2 (ONCII). In multiplex PEA, each target protein is recognized by a pair of proximity probes consisting of an antibody conjugated to a single stranded DNA oligonucleotide that in proximity are hybridized to each other allowing enzymatic DNA polymerization and subsequent DNA amplification [22]. The process creates a signature unique for the specific antigen and quantitatively proportional to the initial concentration of each target protein. The results are obtained as Normalized Protein Expression (NPX) on a log2 scale where a high NPX value corresponds to a high protein concentration. NPX-values were obtained by normalizing values against extension control, negative control that spiked in each sample and a correction factor. Limit of detection (LOD) was determined for each biomarker based on three times standard deviation beyond the NPX value of the negative controls in each run.

The manufacturer of the protein assay, Olink Bioscience, had no influence on the study design, statistical analysis, or manuscript preparation.

The NTpro-BNP and CCL22 measurement in CVDIII did not meet the quality requirements and the results were disregarded, leaving 182 proteins for analysis.

### Analyzes of NTpro-BNP, troponin I and CRP

Since NTpro-BNP is a key cardiovascular protein, supplementary analysis was performed on additional frozen samples at the Department for Clinical Chemistry, Uppsala university hospital together with Troponin I according to standard clinical procedure at the department. CRP was analyzed with standard clinical procedure at the patients’ local laboratory. With CRP included, a total of 185 proteins were analyzed. NTproBNP and Troponin I samples were available for a proportion of patients (133 samples in total).

### Statistics

Categorical variables were expressed as numbers (%) and continuous variables as median (range). All time intervals were measured in months. Association between risk factors and outcome groups were analyzed using Mann-Whitney’s U test, Fisher’s exact test or the Chi square test. Multivariate analysis of risk factors was performed using logistic regression. Missing BMI values were imputed by the median value of the patient group. Overall survival (OS) was analyzed by using the Kaplan-Meier estimator and log-rank test as well as Cox regression.

The difference in protein level between two groups was assessed using linear regression, adjusting for age (at diagnosis), gender and BMI. Significance was determined using linear regression t-test.

To investigate if the protein level or change in protein level over time differ between two groups before, during or after treatment, we studied 87 patients treated with DXR using mixed effects linear regression with protein level as dependent variable, patient id as random effects variable and time point, age, gender, BMI and cardio or CVD or hypertension before as well as the interaction group:time point as fixed effects variables. The association between group and protein level was assessed using a likelihood ratio test. Significant associations were further investigated in post hoc tests.

Benjamini-Hochberg’s false discovery rate method for multiple testing correction was applied and a difference was considered significant if the q-value (the adjusted *p*-value) was ≤0.10.

Statistical analyses were performed with R version 3.6.3 and IBM statistics SPSS version 22.

## Results

### Patients

Clinical characteristics of the 95 patients included in this study are displayed in Table 2. The patient group consisted of 55 (57.9%) men and 40 (42.1%) women with a median age of 65 years (range 27–87). Median follow up time was 69.0 months (range 1–109 months). Seven patients did not receive DXR containing treatment and were only included in base-line analysis. Most patients had 6 cycles of chemotherapy (*n* = 81) resulting in a standard cumulative dose of DXR close to 300 mg/m^2^ (median 292.6 mg/m^2^).

**Table 2:** Demographic and clinical characteristics of 95 eligible patients

Characteristic	Patients n(%)^c^	Missing n(%)
Gender^a^		
Male	55 (57.9)	
Female	40 (42.1)	
Age, years^b^	65.0 (27–87)	
IPI^a^		
0–2	62 (66.7)	2 (2.1)
3–5	31 (33.3)	
B-symtoms^a^		
Yes	32 (33.7)	
No	63 (66.3)	
DXR dose mg/m^2 b^	292.6 (49.1–348.5)^d^	6 (6.3)
Smoking^a^		
Never	39 (52.7)	21 (22.1)
Former	24 (32.4)	
Current	11 (14.9)	
BMI^b^	25.0 (17.5–52.1)	4 (4.2)
eGFR^a^		
0–1	74 (82.2)	5 (5.3)
2–4	16 (17.8)	
CRP^a^		
Normal	32 (34.8)	3 (3.2)
Elevated	60 (65.2)	
Comorbidity^a^		
Heart disease	21 (22.6)	2 (2.1)
Vascular disease	10 (10.8)	2 (2.1)
Hypertension	34 (36.6)	2 (2.1)
DM `	13 (14.0)	2 (2.1)
Medication^a^		
Metformin	9 (9.8)	2 (2.1)
Beta-blocker	18 (19.4)	2 (2.1)
ACE or ARB	26 (28.0)	2 (2.1)
Statins	23 (24.7)	2 (2.1)

Data are presented as ^a^n (%) or ^b^median (range). IPI, international prognostic index; DXR, doxorubicin; BMI, body mass index; eGFR, estimated glomerular filtration rate; CRP, c reactive protein; DM, diabetes mellitus; ACE, angiotensin converting enzyme; ARB, angiotensin II receptor blocker. ^c^ Fever/night sweat/weight loss. ^c^ %; number of patients with X divided with total number of cases with X known. Missing cases are excluded, ^d^Median dose for 82 patients.7 patients recieved no DXR. 6 patients where dose/m2 is missing

### Cardiac disease and CVD

At diagnosis, 21 patients (22.6%) had a pre-existing cardiac condition, 10 (10.8%) vascular disease and 34 (36.6%) hypertension, whereas in the control group, corresponding numbers were 2 (3.3%), 1 (1.7%) and 14 (23.3%). Differences between DLBCL and control group are illustrated in Fig. 1. There was no significant difference in age, gender or BMI between DLBCL and controls, although the DLBCL group had numerally more men (57.9% vs 50%), more individuals > 80 years and with BMI > 35 compared to controls. Fifteen patients got a new diagnosis of a cardiac disease (15.8%) and 22 (23.2%) a new CVD (15 cardiac and 7 vascular) during follow up.

### Survival analysis

Thirty-one patients (32.6%) died during follow up. Causes of death were lymphoma in 20 (64.5%) patients and cardiovascular disease in 4 (12.9%) patients.

**Fig. 2:**
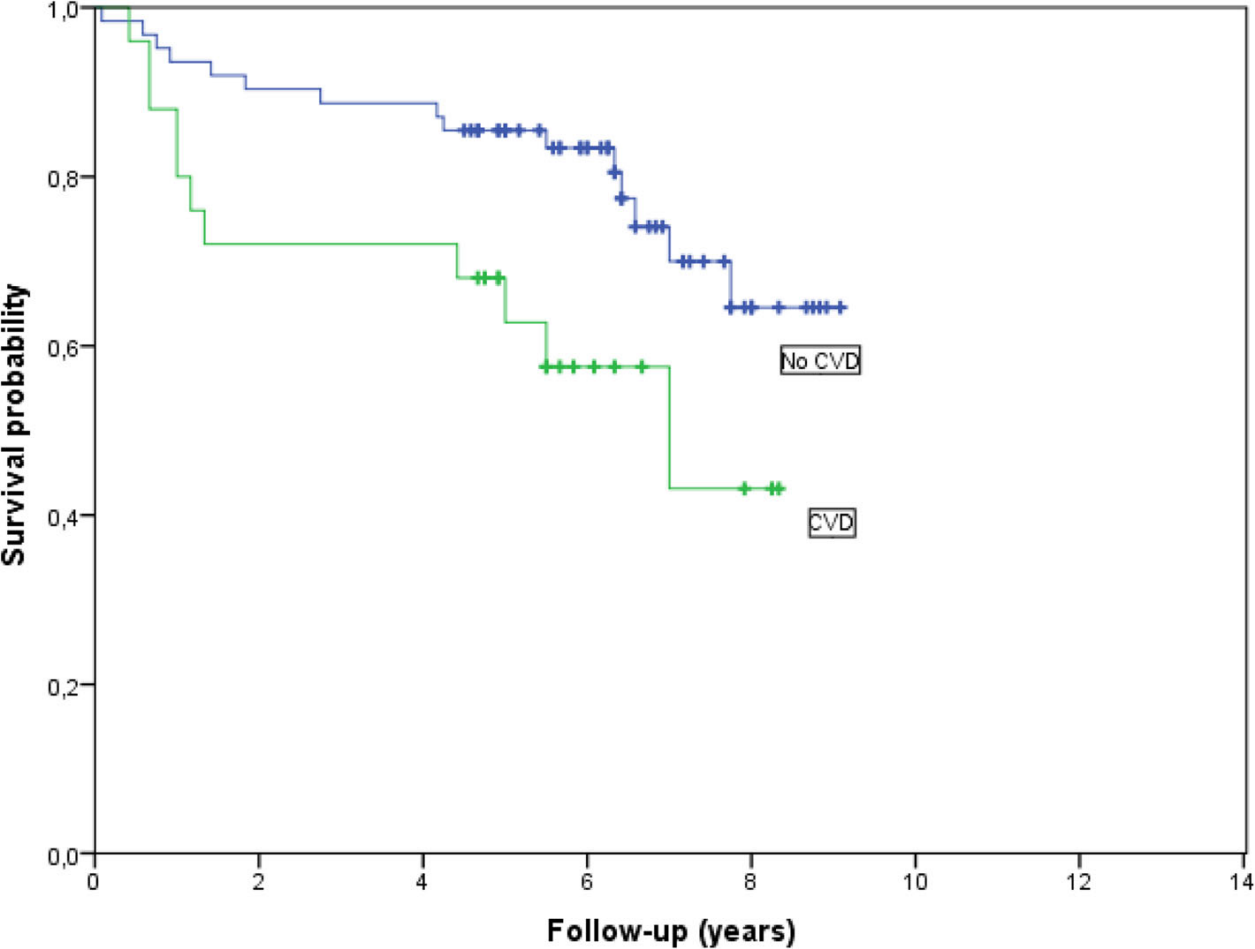
Overall survival survival comparing DXR treated patients (*n* = 87) with CVD (*n* = 25) vs without CVD (*n* = 62) at diagnosis. (*p* = 0.027)

**Fig. 3:**
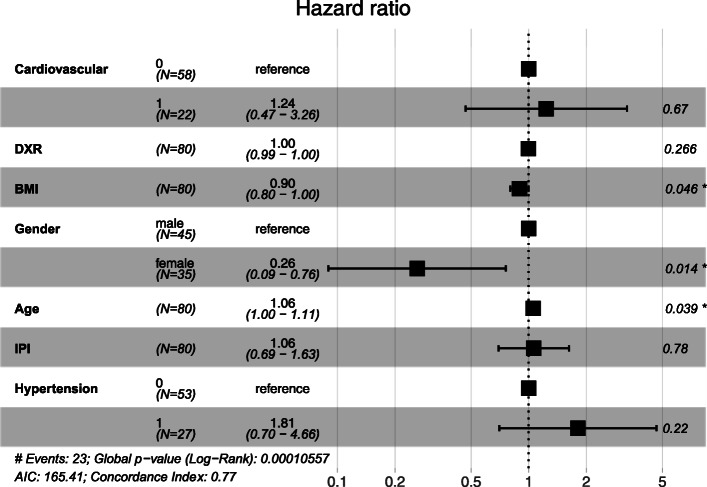
Cox regression analysis of risk factors for overall survival

Patients with pre-existing CVD had a worse OS (*p* = 0.027). Kaplan-Meier curve for OS regarding 87 patients treated with DXR, with or without pre-existing CVD, is shown in Fig. 2. However, Cox-regression analysis including known risk factors for CVD in the 80 cases for whom we had complete data (missing DXR dose, *n* = 5, missing data on hypertension, *n* = 1, missing IPI, n = 1) and imputed missing BMI values (*n* = 4, median value = 25), showed no significant relation for pre-existing CVD and OS. Age (*p* = 0.039), gender (*p* = 0.014) and BMI (*p* = 0.046) were associated to OS (Fig. 3).

### Protein analysis

We found no proteins in the PEA-CO or NTproBNP and Troponin I in DLBCL pre-treatment samples that significantly correlated with pre-existing cardiac disease. For NTproBNP, analyzed in 29 samples prior to treatment, the non-adjusted *p*-value was 0.002 and adjusted q = 0.43. However, in patients with pre-existing CVD there was an association between higher levels of SPON-1 and CVD at diagnosis as estimated from 92 PEA-CO samples (Fold change (FC) 1.22, 95%CI 1.10–1.35, *p* = 0.0002, q = 0.046).

We observed a significant association between higher level of protein IL-1RT1 in pre-treatment samples and upcoming CVD (adjusted for previous CVD and hypertension, 92 samples) (FC 1.24, 95%CI 1.10–1.39, *p* = 0.0004, q = 0.082).

**Fig. 4:**
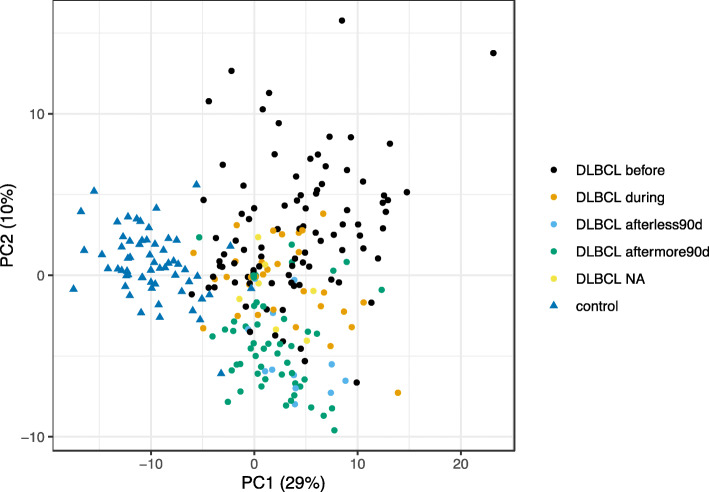
PCA protein patterns for controls and DLBCL

**Fig. 5:**
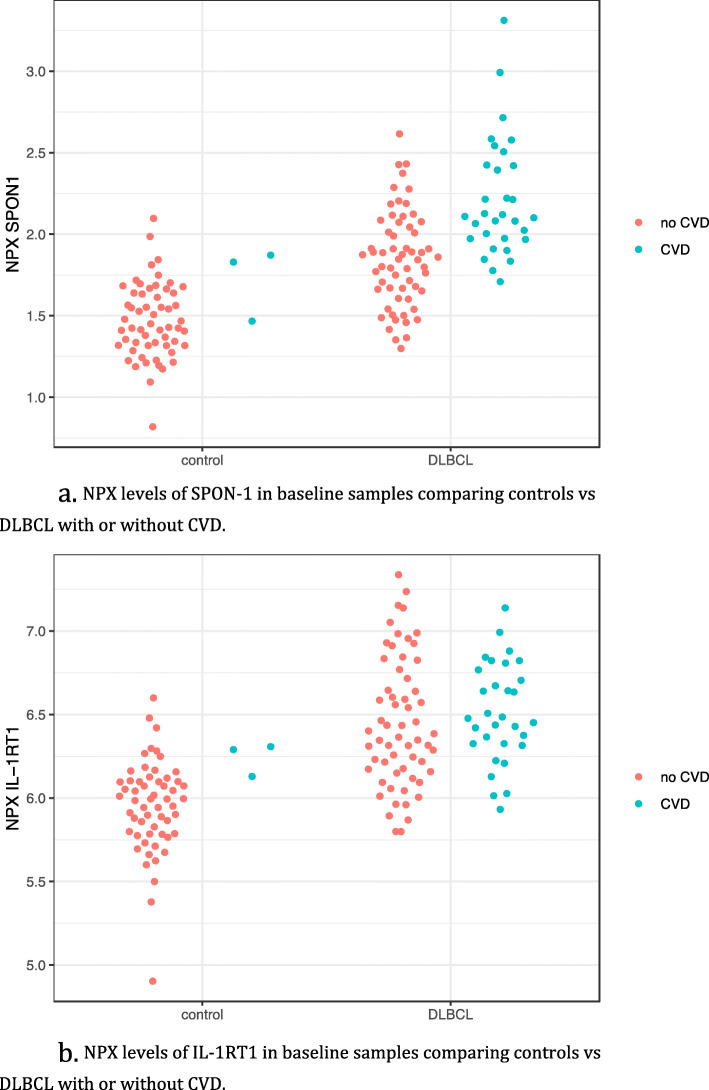
a. NPX levels of SPON-1 in baseline samples comparing controls vs DLBCL with or without CVD. b. NPX levels of IL-1RT1 in baseline samples comparing controls vs DLBCL with or without CVD

Comparison of baseline protein patterns between DLBCL and controls revealed pronounced differences in many proteins illustrated in Fig. 4. The differences were not analysed further since it was not the scope of this study. Furthermore, there were only three cases with CVD in the control cohort why differences between protein levels in relation to CVD between controls and patients not could be analyzed. For the separate proteins of interest we found a difference in levels of SPON-1 and IL-1RT1 between DLBCL and controls regardless of existing CVD or not (Fig. 5a, b).

We investigated if treatment affect the protein levels differently in patients developing a new cardiac disease or CVD as compared to patients who did not develop cardiac disease or CVD.

**Fig. 6:**
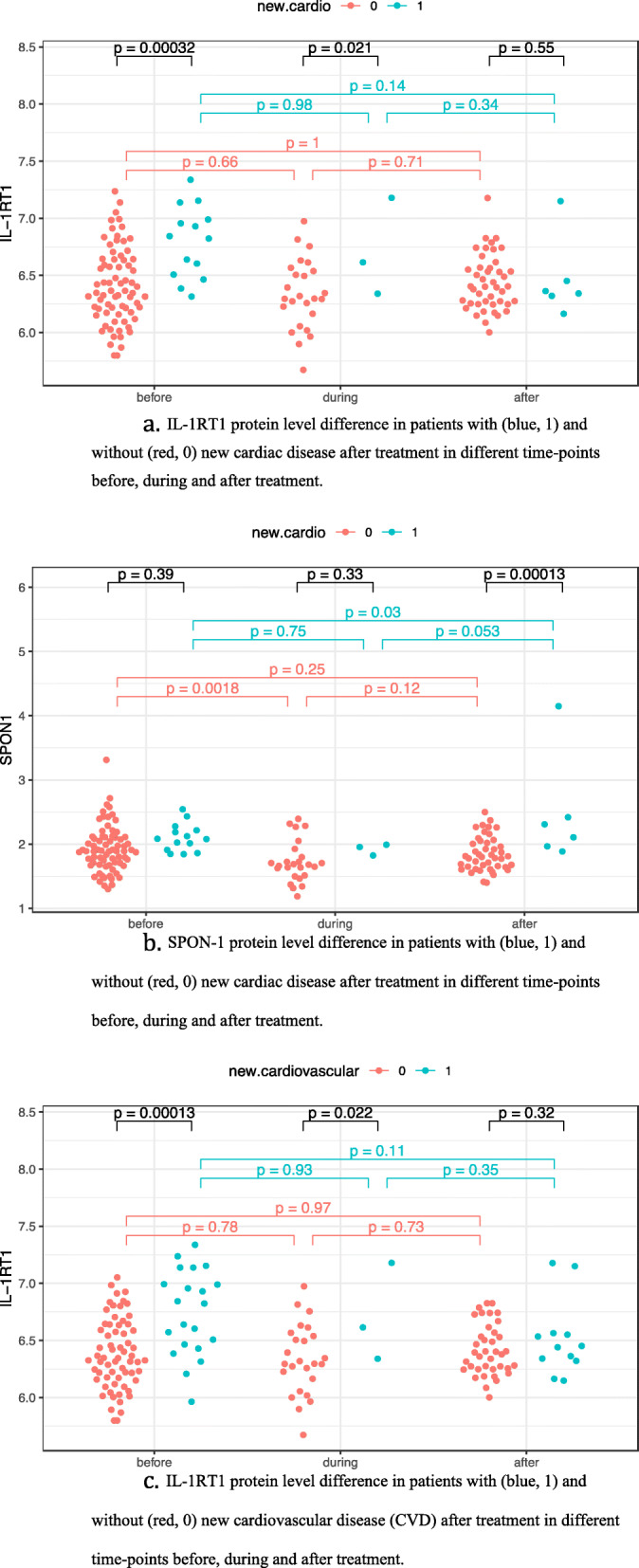
a. IL-1RT1 protein level difference in patients with (blue, 1) and without (red, 0) new cardiac disease after treatment in different time-points before, during and after treatment. b. SPON-1 protein level difference in patients with (blue, 1) and without (red, 0) new cardiac disease after treatment in different time-points before, during and after treatment. c. IL-1RT1 protein level difference in patients with (blue, 1) and without (red, 0) new cardiovascular disease (CVD) after treatment in different time-points before, during and after treatment

There was a significant difference related to new cardiac disease for IL-1RT1 (*p* = 0.007, q = 0.096) and SPON-1 (*p* = 0.001, q = 0.096). Post hoc analyses show that for IL-1RT1 this is due to a difference in protein level between patients with and without new cardiac disease mainly before, but also during treatment (Fig. 6a). The difference in SPON1 is due to a group difference after treatment. (Fig. 6b). Emerging CVD was associated with change in IL-1RT1 (*p* = 0.0003, q = 0.056). Post hoc analysis to clarify time point for this protein level change revealed a difference between samples for new CVD vs no new CVD before and during treatment but not for samples after (Fig. 6c).

Univariate tests for suspected CVD risk factors and drugs with possible association to CVD came up with one significant factor; age (*p* = 0.003 q = 0.057) pointing at a higher risk for CVD and three suspected risk factors: treatment with angiotensin converting enzyme/angiotensin II receptor (ACE/ARB) blockers (*p* = 0.025, q = 0.228), DXR (*p* = 0.121, q = 0.436) and hypertension (*p* = 0.105, q = 0.436) probably connected with CVD (Table 3). All risk factors with a *p*-value below 0.20 were combined in a multivariate logistic regression, in which no factor showed significant association to CVD.

**Table 3:** Univariate tests for suspected CVD risk factors and drugs with possible association to CVD

Variable	No CVD^b^	CVD^c^	Test^d^	p-value	q-value
Number^a^	71	22			
Age (mean)	61.4	71.1	Mann-W	0.003	0.057
Gender = 1(%)	32 (45.1)	7 (31.8)	Chisq	0.393	0.885
DXR (mean)	493.4	417.9	Mann-W	0.121	0.436
Smoking (%)	24 (43.6)	9 (52.9)	Chisq	0.693	0.960
BMI (mean)	26.3	24.7	Mann-W	0.282	0.726
Cardiac (%)	14 (20.0)	5 (23.8)	Chisq	0.944	1.000
CVD (%)	21 (30.0)	8 (38.1)	Chisq	0.666	0.960
HT (%)	21 (30.0)	11 (52.4)	Chisq	0.105	0.436
DM(%)	9 (12.9)	4 (19.0)	Fisher	0.488	0.960
CRP (mean)	40.2 (70.9)	39.1 (53.6)	Mann-W	0.955	1.000
GFR (%)	11 (16.2)	5 (23.8)	Chisq	0.638	0.960
AB (%)	26 (36.6)	6 (27.3)	Chisq	0.583	0.960
IPIhigh (%)	22 (31.9)	7 (31.8)	Chisq	1.000	1.000
Metformin = 1(%)	7 (10.0)	2 (9.5)	Fisher	1.000	1.000
B-block (%)	10 (14.3)	6 (28.6)	Chisq	0.237	0.712
ACE/ARB (%)	14 (20.0)	10 (47.6)	Chisq	0.025	0.228
B-block/ACE-ARB (%)	19 (27.1)	11 (52.4)	Chisq	0.058	0.350
Statin (%)	16 (22.9)	6 (28.6)	Chisq	0.806	1.000

Gender = 1, females; DXR,doxorubicin; Smoking, ongoing or earlier smoker; BMI, body mass index; Cardiac, cardiac disease at diagnosis, CVD, cardiovascular disease at diagnosis; HT, hypertension at diagnosis; DM, diabetes mellitus at diagnosis; CRP, c-reactive protein; GFR, glomerular filtration rate < 60 ml/min/1.72m^2^; AB, B-symtoms (fever, night sweats, weight loss) at diagnosis; IPIhigh, International prognostic index score ≥ 3; B-block, treatment with beta-blocker; ACE/ARB, treatment with angiotensine converting enzyme/ angiotensine II receptor blocker; Statin, treatment with statins

Mann-W, Mann-Whitney test; Chisq, Chisquare test; Fisher, Fishers exact test

^a^ 93 patients included. 2 patients excluded because of lack of information on emerging CVD

^b^No new CVD after treatment

^c^Emerging CVD after treatment

^d^type of test performed

## Discussion

In our study, we found that high SPON-1 levels are associated with existing CVD in DLBCL patients. Our results are, to best of our knowledge, the first to reveal this relation between SPON-1 and CVD in DLBCL patients. SPON-1 is a cell adhesion protein important for axons and a major factor for vascular smooth muscle cell activity, which might explain the importance in cardiac toxicity (www.uniprot.org). In recent studies on patients with [34] or without [26, 35] pre-existing hearth failure, SPON-1 was associated with incident or deteriorating heart failure. Interestingly, in one of the studies, chronic kidney disease was significantly interacting with the association, something that would be of concern to investigate further in our cohort [35].

The Interleukin-1 receptor type 1, IL-1RT1, was associated with the risk of developing cardiovascular disease. IL-1RT1 is a receptor for IL1A, IL1B and IL1RN which after binding mediates activation of NF-kappa-beta, MAPK and other pathways (www.uniprot.org). IL-1 blockade can reduce myocardial infarct size and injury by interrupting the inflammatory reaction even after DXR exposure in animal studies [36, 37].

Previous studies using the Olink proteomic technology on deterioration of, or emerging cardiac disease, have shown divergent results. There are findings of a range of separate proteins associated with heart failure but no correlation between the different proteins associated in the separate studies [23–27]. One study have reported the association of SPON-1 and incident heart failure in two community-based prospective cohorts of elderly without heart failure at baseline [26], further strengthening the implications of SPON-1 in CVD.

To our knowledge, there are no reports on association between IL-1RT1 levels in plasma and heart toxicity in humans and our results might be the first to demonstrate a possible association between plasma IL-1RT1 levels and upcoming CVD after DXR treatment in humans.

Since PEA technology is new and not exact it would be of great interest to support and validate the results with established technologies such as ELISA. Unfortunately, there were no remaining samples from the included cases to do this in our study.

NTproBNP has in several studies been shown to be suitable as a quantitative plasma biomarker for the diagnosis of heart failure [16]. In chronic heart failure, the level and pattern of increase in NTproBNP, Troponin T and CRP is shown to be associated with adverse prognosis. On the contrary, studies on DXR treated cancer patients has only shown correlation between Troponin I level and cardiac toxicity independent from NTproBNP [17–19]. In the present study, neither NTproBNP nor Troponin I significantly covaried with present or emerging cardiac or cardiovascular disease. The low sample frequency, of NTproBNP and Troponin I as well as the limited sample size may explain the lack of correlation, although NTproBNP was the protein with the closest to significant level on association with present cardiac disease (q = 0.43).

There was a large difference in many protein levels between DLBCL and controls regardless of CVD including SPON-1 and IL-1RT1 and one might speculate that those levels is a reflection of the inflammatory state of the DLBCL patients.

We describe a high proportion of patients with pre-existing CVD before treatment initiation, compared to a non DLBCL cohort, a finding not previously described to our knowledge. However, the number of controls were low and not completely matched and the finding must be interpreted with caution. In Sweden, the prevalence of ischemic heart disease in the population had been reported to be 3.858/100.000 which corresponds to 3.86% [38]. This number is similar to the prevalence in our control cohort (3.3%), making the almost seven times higher prevalence of 22.6% in the DLBCL group remarkable. The occurrence of hypertension in the Swedish general population is not fully mapped, but data from the public health agency of Sweden (www.folkhalsomyndigheten.se) and the Swedish association of local authorities and regions (www.vardhandboken.se) indicate a prevalence of around 20–30%, which is similar to the frequency as in our control group (23.3%). Again, higher prevalence was seen in our DLBCL cohort (36.6%, *p* = 0.063).

In a recent study of chronic lymphocytic leukemia (CLL), a prevalence of CVD diagnosis, including hypertension, within 10 years prior to CLL, was found to be 32% [39] which is similar to our study. A possible relation between lymphatic malignancies i.e. common risk factors or an inflammatory state increasing the risk of lymphoma might be speculated on and of interest to explore in future studies.

In this study, we report a high incidence of emerging CVD, where almost one fourth (23.2%) of DLBCL patients receiving immunochemotherapy develop CVD. Previous studies show an incidence of emerging heart toxicity of 10–20% for AC or DXR treated cancer and lymphoma patients [17, 18, 21, 40–43]. Our result of 15.9%, with a quite long follow up time, fits into these results, despite the fact that our cases were clinically evident disease, whereas in some studies cases of silent heart failure only observed in ultrasound measurements were also included [21, 40, 41]. Some of the mentioned studies have a rather low median age [41, 42] and our older, population based cohort would be expected to be more prone to cardiac and cardiovascular diseases.

Suspected risk factors for emerging CVD were age, DXR dose, hypertension, and ACE/ARB treatment. None of them was significant in our multivariate testing probably due to covariation of these factors (at least for age, hypertension, and ACE/ARB usage). Although it is plausible that other factors such as DM, renal function (GFR), BMI and smoking can be of importance, we could not observe such associations which might be due to missing data in patients records and the small cohort.

## Conclusion

In conclusion, our study revealed two new proteins, SPON-1 and IL-1RT1, possibly related to pre-existing and emerging CVD respectively in DLBCL patients treated with DXR. If confirmed in larger study cohorts, IL-1RT1 may emerge as a very promising biomarker for the increased risk of developing CVD in DLBCL patients. In addition, we observed higher prevalence of cardiac disease and CVD in DLBCL patients compared to the average population. Larger epidemiological studies may confirm these results and possibly unravel the relation between CVD and DLBCL development – further studies are required to elucidate whether CVD increases the risk of DLBCL or vice versa. In clinical practice our results points out the importance of exerting caution in caring for DLBCL patients with CVD and the importance of considering preventive strategies and eventually optimizing therapy for CVD in these patients.

There are limitations in this study. First, the cohort is relatively small and missing values for NTproBNP and Troponin I proteins at start were high. There were to few samples during and after treatment to make any sure assumptions on the results. We could not validate the protein analyses with established technologies due to lack of remaining samples. Second, the diagnoses are only based on revision of medical records, not by any additional investigations (e.g. cardiac ultrasound) making the characterization of disease unsure and the time for evolvement of new cardiac or vascular after treatment is unknown, Furthermore the follow-up time is too short to evaluate long term toxicities.

## Supplementary Information

**Additional file 1.** Supplementary Table 1. List of proteins included in CVDIII and ONCII panels.

**Additional file 2.**

**Additional file 3.**

## Data Availability

The datasets used and/or analysed during the current study are available from the corresponding author on reasonable request.
