# Supplementary material for: Plasma proteome profiling of cardiotoxicity in patients with diffuse large B-cell lymphoma
Source: Cardiooncology. 2021 Feb 3;7:6. doi: 10.1186/s40959-021-00092-0 (PMC7856776; doi:10.1186/s40959-021-00092-0)
Supplement: Supplementary file 1 — Additional file 1. Supplementary Table 1. List of proteins included in CVDIII and ONCII panels. [file 40959_2021_92_MOESM1_ESM.docx]

Supplementary Table 1.

List of proteins included in CVDIII and ONCII panels

|  |  |  |  |
| --- | --- | --- | --- |
| **CVDIII** |  | **ONCII** |  |
| *Protein* | *Full name* | *Protein* | *Full name* |
|  |  |  |  |
| AP-N | Aminopeptidase N | 5’-NT | 5’-nucleotidase |
| AZU1 | Azurocidin | ADAM-TS 15 | A disintegrin and metalloproteinase with thrombospondin motifs 15 |
| BLM hydrolase | Bleomycin hydrolase | TXLNA | Alpha-taxilin |
| CCL15 | C-C motif chemokine 15 | AREG | Amphiregulin |
| CCL16 | C-C motif chemokine 16 | ANXA1 | Annexin 1 |
| CCL24 | C-C motif chemokine 24 | CD207 | C-type lectin domain family 4 member K |
| CXCL16 | C-X-C motif chemokine 16 | CXCL13 | C-X-X motif chemokine 13 |
| CDH5 | Cadherin-5 | CAIX | Carbonic anhydrase IX |
| CPA1 | Carboxypeptidase A1 | CPE | Carboxypeptidase E |
| CPB1 | Carboxypeptidase B | CEA | Carcinoembryonic antigen |
| CASP-3 | Caspase-3 | CEACAM1 | Carcinoembryonic antigen-related cell adhesion molecule 1 |
| CTSD | Cathepsin D | CTSV | Cathepsin L2 |
| CTSZ | Cathepsin Z | CD160 | CD 160 antigen |
| ALCAM | CD166 antigen | CD27 | CD27 antigen |
| CHI3L1 | Chitinase-3-like protein 1 | CD48 | CD48 antigen |
| CHIT1 | Chitotriosidase-1 | CD70 | CD70 antigen |
| COL1A1 | Collagen alpha-1(I) chain | CRNN | Cornulin |
| CD93 | Complement component C1q receptor | CDKN1A | Cyclin-dependent kinase inhibitor 1 |
| CNTN1 | Contactin-1 | DLL1 | Delta-like protein 1 |
| CSTB | Cystatin-B | ADAM8 | Disintegrin and metalloproteinase domain-containing protein 8 |
| SELE | E-selectin | ESM-1 | Endothelial cell-specific molecule 1 |
| P13 | Elafin | EPHA2 | Ephrin type-A receptor 2 |
| EPHB4 | Ephrin type-B receptor | FasL | Fas antigen ligand |
| EGFR | Epidermal growth factor | FADD | FAS-associated death domain protein |
| Ep-CAM | Epithelial cell adhesion molecule | FCRLB | Fc receptor-like B |
| FABP4 | Fatty acid-binding protein, adipocyte | FGF-BP1 | Fibroblast growth factor-binding protein 1 |
| Gal-3 | Galectin-3 | FR-alpha | Follate receptor alpha |
| Gal-4 | Galectin-4 | FR-gamma | Folate receptor gamma |
| GRN | Granulins | FUR | Furin |
| GDF-15 | Growth/differentiation factor 15 | Gal-1 | Galectin-1 |
| IGFBP-1 | Insuline-like growth factor-binding protein 1 | GPC1 | Glypican-1 |
| IGFBP-2 | Insuline-like growth factor-binding protein 2 | GZMB | Granzyme B |
| IGFBP-7 | Insuline-like growth factor-binding protein 7 | GZMH | Granzyme H |
| ITGB2 | Integrin beta-2 | HGF | Hepatocyte growth factor |
| ICAM-2 | Intercellular adhesion molecule 2 | ICOSLG | ICOSligand |
| IL-1RT1 | Interleukin-1 receptor type 1 | IGF1R | Insuline-like growth factor 1 receptor |
| IL-1RT2 | Interleukin-1 receptor type 2 | ITGAV | Integrin alpha-V |
| IL-17RA | Interleukin-17 receptor A | ITGB5 | Integrin beta-5 |
| IL-18BP | Interleukin-18-binding protein | IFN-gamma-R1 | Interferon gamma receptor 1 |
| IL2-RA | Interleukin-2 receptor subunit alpha | IL6 | Interleukin-6 |
| IL-6RA | Interleukin-6 receptor subunit alpha | hK11 | Kallikrein 11 |
| JAM-A | Junctional adhesion molecule A | KLK13 | Kallikrein 13 |
| KLK6 | Kallikrein-6 | hK14 | Kallikrein-14 |
| LDL receptor | Low-density lipoprotein receptor | hK8 | Kallikrein-8 |
| LTBR | Lymphotoxin-beta receptor | LYPD3 | Ly6/PLAUR domain-containing protein 3 |
| MEPE | Matrix extracellular phosphoglycoprotein | MIA | Melanoma-derived growth regulatory protein |
| MMP-2 | Matrix metalloproteinase-2 | MSLN | Mesothelin |
| MMP-3 | Matrix metalloproteinase- | MetAP2 | Methionine aminopeptidase 2 |
| MMP-9 | Matrix metalloproteinase-9 | MIC-A/B | MHC class I polypeptide-related sequence A/B |
| TIMP4 | Metalloproteinase inhibitor 4 | MK | Midkine |
| MCP-1 | Monocyte chemotactic protein 1 | MAD homolog 5 | Mothers against decapentaplegic homolog 5 |
| PRTN3 | Myeloblastin | MUC-16 | Mucin-16 |
| MPO | Myeloperoxidase | PVRL4 | Nectin-4 |
| MB | Myoglobin | PPY | Pancreatic prohormone |
| NT-proBNP | N-terminal prohormone brain natriuretic peptide | PODXL | Podocalyxin |
| Notch 3 | Neurogenic locus notch homolog protein 3 | EGF | Pro-epidermal growth factor |
| OPN | Osteopontin | CYR61 | Protein CYR61 |
| OPG | Osteoprotegerin | S100A11 | Protein S100-A11 |
| SELP | P-selectin | S100A4 | Protein S100-A4 |
| PON3 | Paraoxonase | RET | Proto-oncogene tyrosine-protein kinase receptor Ret |
| PGLYRP1 | Peptidoglycan recognition protein 1 | RSPO3 | R-spondin-3 |
| PLC | Perlecan | ErbB2/HER2 | Receptor tyrosine-protein kinase erbB-2 |
| PAI | Plasminogen activator inhibitor | ErbB3/HER3 | Receptor tyrosine-protein kinase erbB-3 |
| PECAM-1 | Platelet endothelial cell adhesion molecule | ErbB4/HER4 | Receptor tyrosine-protein kinase erbB-4 |
| GP6 | Platelet glycoprotein VI | SCAMP3 | Secretory carrier-associated membrane protein 3 |
| PDGF subunit A | Platelet-derived growth factor subunit A | SEZ6L | Seizure 6-like protein |
| PCSK9 | Proprotein convertase subtilisin/kexin type 9 | SPARC | SPARC |
| DLK-1 | Protein delta homolog 1 | SCF | Stem cell factor |
| PSP-D | Pulmonary surfactant-assocoated protein D | SYND1 | Syndecan-1 |
| RETN | Resistin | TCL1A | T-cell leukemia/lymphoma protein 1A |
| RARRES2 | Retinoic acid receptor responder protein 2 | LY9 | T-lymphocyte surface antigen Ly-9 |
| CD163 | Scavenger receptor cysteine-rich type 1 protein | TGFR-2 | TGF-beta receptor type-2 |
| SCGB3A2 | Secretoglobin family 3A member 2 | TFP1-2 | Tissue factor pathway inhibitor 2 |
| SPON1 | Spondin-1 | TRAIL | TNF-related apoptosis-including ligand |
| ST2 | ST2 protein | TLR3 | Toll-like receptor |
| TR-AP | Tartrate-resistant acid phosphatase type 5 | TGF-alpha | Transforming growth factor alpha |
| TFP1 | Tissue factor pathway inhibitor | GPNMB | Transmembrane glycoprotein NMB |
| t-PA | Tissue-type plasminogen activator | TNFSF13 | Tumor necrosis factor ligand superfamily member 13 |
| TR | Transferrin receptor protein 1 | TNFRSF19 | Tumor necrosis factor receptor superfamily member 19 |
| TFF3 | Trefoil factor 3 | TNFRSF4 | Tumor necrosis factor receptor superfamily member 4 |
| TLT-2 | Trem-like transcript 2 protein | TNFRSF6B | Tumor necrosis factor receptor superfamily member 6B |
| TNFSF13B | Tumor necrosis factor ligand superfamily member 13B | ABL1 | Tyrosine-protein kinase ABL1 |
| TNF-R1 | Tumor necrosis factor receptor 1 | LYN | Tyrosine-protein kinase Lyn |
| TNF-R2 | Tumor necrosis factor receptor 2 | VEGF-A | Vascular endotelial growth factor A |
| TNFRSF10C | Tumor necrosis factor receptor superfamily member 10C | VEGFR-2 | Vascular endotelial growth factor receptor 2 |
| TNFRSF14 | Tumor necrosis factor receptor superfamily member 14 | VEGFR-3 | Vascular endotelial growth factor receptor 3 |
| FAS | Tumor necrosis factor receptor superfamily member 6 | CXCL17 | VEGF-co regulated chemokine 1 |
| AXL | Tyrosine-protein kinase receptor UFO | VIM | Vimentin |
| SHPS-1 | Tyrosine-protein phosphatase non-receptor type substrate 1 | WFDC2 | WAP four-disulfide core domain protein 2 |
| U-PAR | Urokinase plasminogen activator surface receptor | WIF-1 | Wnt inhibitory factor 1 |
| uPA | Urokinase-type plasminogen activator | WISP-1 | WNT1-inducible-signaling pathway protein 1 |
| vWF | Von Willebrand factor | XPNPEP2 | Xaa-Pro aminopeptidase 2 |
