# Supplementary material for: Plasma proteome profiling of cardiotoxicity in patients with diffuse large B-cell lymphoma
Source: Cardiooncology. 2021 Feb 3;7:6. doi: 10.1186/s40959-021-00092-0 (PMC7856776; doi:10.1186/s40959-021-00092-0)
Supplement: Supplementary file 2 — Additional file 2. [file 40959_2021_92_MOESM2_ESM.pdf]

1. Introduction

Olink® CARDIOVASCULAR III is a reagent kit measuring 92 cardiovascular disease (CVD)--related human protein biomarkers simultaneously. The assays on this panel have been selected to focus on high-abundance proteins, and 1 µL of a 1:100 dilution of sample is used. The analytical performance of the product has been carefully validated and the results are presented in this document. Please note that when a new panel is developed, both the individual assays and 92-plex panel as a whole are subject to our thorough validation procedure. If individual assays are subsequently improved or one or more assays are replaced in later versions of the panel, focus is placed on thoroughly validating the individual assays in question.

1.1 TECHNOLOGY

The Olink reagents are based on the Proximity Extension Assay (PEA) technology[1-2], where 92 oligonucleotide labeled antibody probe pairs are allowed to bind to their respective target proteins, if present in the sample. A PCR reporter sequence is formed by a proximity-dependent DNA polymerization event. This is then amplified, and subsequently detected and quantified using real-time PCR. The assay is performed in a homogeneous 96-well format without any need for washing steps, see Figure 1.

1.2 QUALITY CONTROLS

Internal and external controls have been developed by Olink for data normalization and quality control purposes. These controls are designed to enable monitoring of the technical assay performance, as well as the quality of individual samples, and provide information at each step of the Olink protocol (see Figure 1). The internal controls are added to each sample and include two Immunoassay controls, one Extension control and one Detection control. The Immunoassay controls (two non-human proteins) monitor all three steps starting with the immunoreaction. The Extension Control (an antibody linked to two matched oligonucleotides) monitors the extension and readout steps independent of antigen binding, and is used for data normalization across samples. Finally, the Detection control (a synthetic double-stranded template) monitors the readout step. Samples for which one or more of the internal control values deviate from a pre-determined range will be flagged and may be removed before statistical analysis. An external inter-plate control (IPC), is included on each plate and is used in a second normalization step. This control is made up of a pool of probes similar to the Extension control (Ext Ctrl), but generated with 92 matching oligonucleotide pairs. This improves inter-assay precision and allows for optimal comparison of data derived from multiple runs. The term "Normalized Protein eXpression (NPX)"refers to normalized data as described above.

1.3 DATA ANALYSIS

Data analysis is performed by employing a pre-processing normalization procedure. For each sample and data point, the corresponding Cq-value for the Extension control is substracted, thereby normalizing for technical variation within one run. Normalization between runs is then performed for each assay by substracting the corresponding dCq-value for the Interplate Control (IPC) from the dCq-values generated. In the final step of the pre-processing procedure the values are set relative to a correction factor determined by Olink. The Normalized Protein eXpression (NPX) unit is generated on a log2 scale where a larger number represents a higher protein level in the sample, typically with the background level at around zero. Linearization of data is performed by the mathematical operation $2^{\wedge NPX}$. Coefficient of variation (CV) calculations are performed on linearized values.

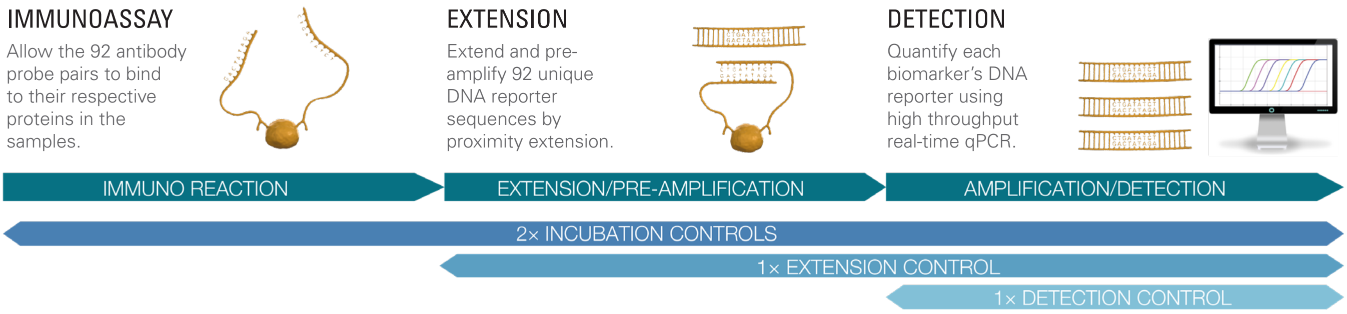

Fig 1. Olink assay procedure (above) and controls (below). The internal controls enable monitoring of the three core steps in the Olink assay and used for quality control and data normalization. Detection is performed by using the Fluidigm® Biomark™ or the Fluidigm® Biomark™ HD system.

2. Performance characteristics

2.1 SAMPLE TYPES

Performance with different sample types was evaluated for Olink CARDIOVASCULAR III by collecting matched EDTA-, acid citrate dextrose (ACD)- and sodium heparin-plasma, as well as serum samples from 4 healthy individuals. Comparative response values between heparin plasma, citrate plasma or serum, are expressed as relative differences (%) compared to EDTA plasma and are shown in Table 1 for each sample type. To evaluate the measuring range for endogenous protein levels, response values levels were assessed in 20 normal EDTA plasma samples and reported in NPX (Table 1). Variations observed between responses in heparin and citrate plasma, as compared to EDTA plasma, were generally small, and most of the assays will therefore function without any limitations related to the anti-coagulant used.

2.2 ANALYTICAL MEASUREMENT

NOTE: *The technical performance data based on in vitro assays using recombinant antigen must* **NOT** *be used to derive actual concentrations of native proteins in biological samples from the relative quantification NPX data that is obtained from an Olink assay.*

DETECTION LIMIT

Calibrator curves were determined for all biomarkers simultaneously in a multiplex format. Limit of detection (LOD) was defined as 3 standard deviations above background and reported in pg/mL for all assays for which recombinant protein antigen was available, see Table 1 and Figure 2.

HIGH DOSE HOOK EFFECT

High dose hook effect is a state of antigen excess relative to the reagent antibodies resulting in falsely lower values. In such cases a significantly lower value can be reported which leads to misinterpretation of results. Therefore, the hook effect was determined for each analyte, here reported in pg/mL for all 92 assays, see Table 1.

MEASURING RANGE

The analytical measuring range was defined by the lower limit of quantification (LLOQ) and upper limit of quantification (ULOQ) and reported in pg/mL. Quantification limits of LLOQ and ULOQ were calculated with the following trueness and precision criteria; relative error ≤ 30% and CV ≤ 30%, of back-calculated values, respectively. Measuring ranges were reported in order of log10, see Table 1.

Three assays with their analytical data are exemplified in Figure 2 and the distribution of measuring ranges of 92 assays and endogenous plasma levels is shown in Figure 3. Separate calibrator curves established for each assay may be viewed at www.olink.com/cvd3.

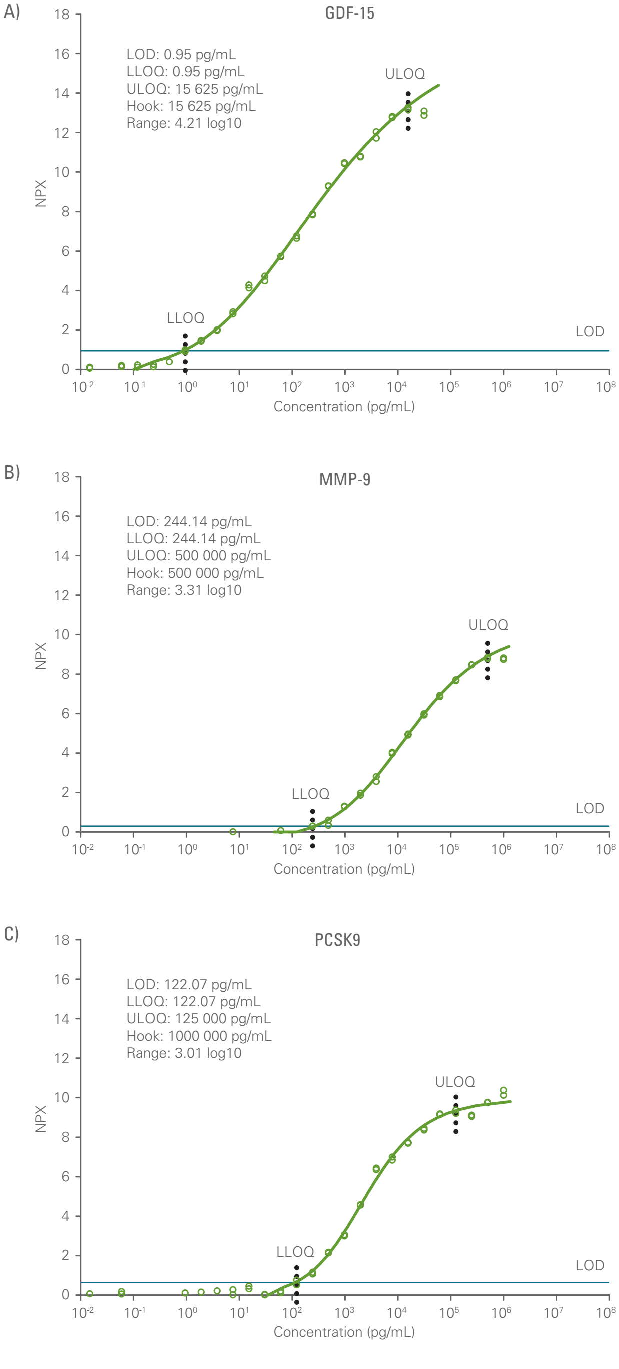

Fig 2. Calibrator curves for representative assays using a 4-parameter curve fitting model.

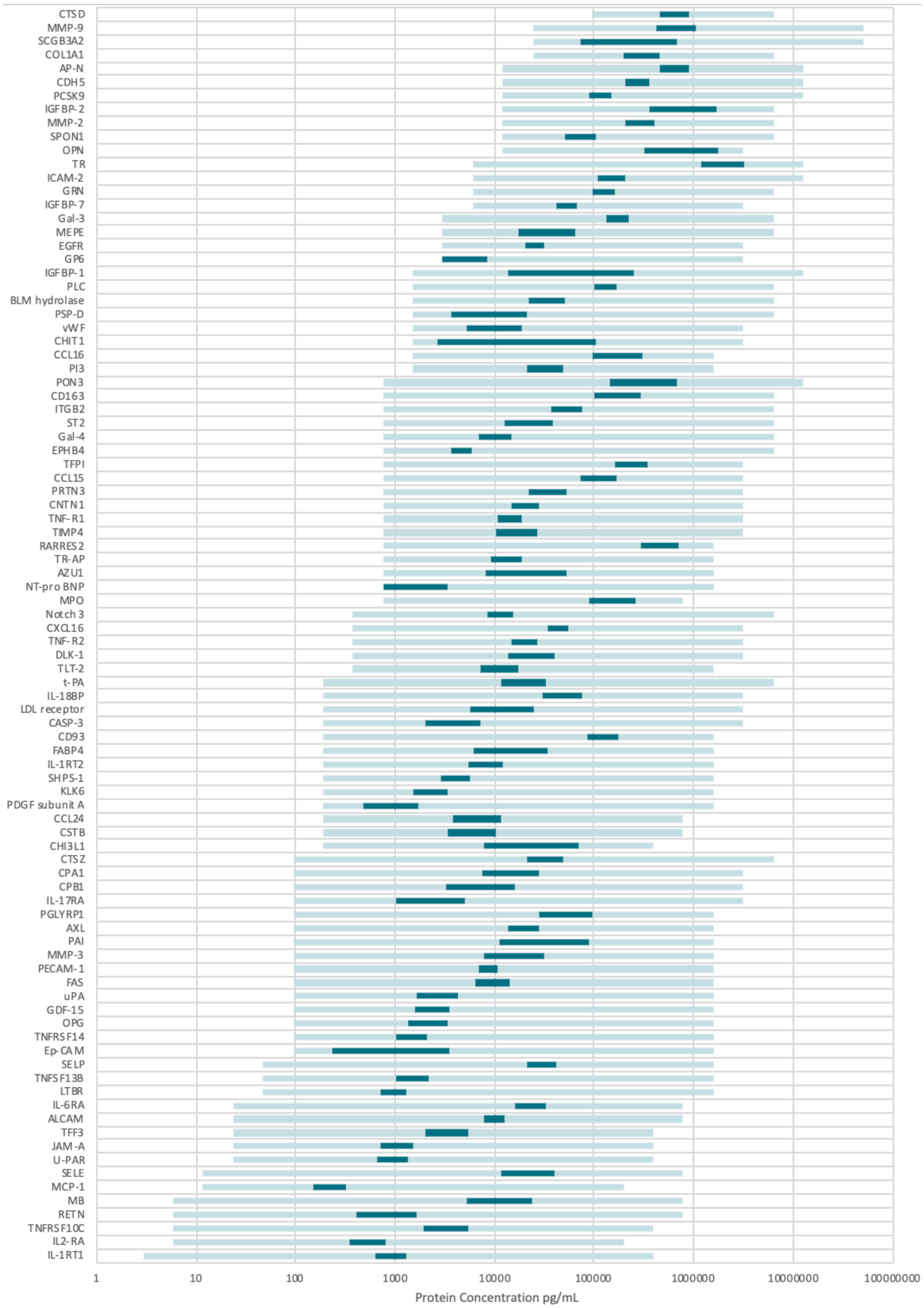

Fig 3. Distribution of analytical measuring range, defined by the lower and upper limits of quantification (LLOQ-ULOQ), and normal plasma levels where data is available (dark blue bars) for 92 analytes.

Table 1. Sample Types; Normalized Protein eXpression (NPX), Endogenous Interference, Analytical Measurement; Limit of Detection (LOD), Lower Limit of Quantification (LLOQ), Upper Limit of Quantification (ULOQ), High Dose Effect (Hook), Range and Precision indicative of assay performance are shown for 92 analytes. Not available, NA

		Sample types						Endogenous interference	Analytical measurement					Precision	
		Percentile EDTA plasma (NPX)			Relative to EDTA plasma (%)			mg/mL	pg/mL				log10	% CV	
Target	UniProt No	10th	Median	90th	ACD	Heparin	Serum	Haemolysate	LOD	LLOQ	ULOQ	Hook	Range	Intra	Inter
Aminopeptidase N (AP-N)	P15144	5.1	5.5	6	100	101	100	15	61.04	122.07	125 000	125 000	3	7	8
Azurocidin (AZU1)	P20160	2.2	2.9	4.6	84	509	979	0.5	7.63	7.63	15 625	31 250	3.3	7	8
Bleomycin hydrolase (BLM hydrolase)	Q13867	4.7	5.1	5.8	82	84	97	0.5	7.63	15.26	62 500	62 500	3.6	8	11
Cadherin-5 (CDH5)	P33151	3.9	4.4	4.9	108	115	108	15	122.07	122.07	125 000	125 000	3	11	12
Carboxypeptidase A1 (CPA1)	P15085	3.8	4.6	5.6	102	105	111	15	0.95	0.95	31 250	62 500	4.5	7	10
Carboxypeptidase B (CPB1)	P15086	3.5	4.4	5.7	90	93	96	15	0.95	0.95	31 250	62 500	4.5	7	12
Caspase-3 (CASP-3)	P42574	4.3	4.9	6.2	63	53	44	0	1.91	1.91	31 250	62 500	4.2	9	16
Cathepsin D (CTSD)	P07339	3.2	3.5	4.3	93	100	115	15	976.56	976.56	62 500	125 000	1.8	7	10
Cathepsin Z (CTSZ)	Q9UBR2	4.5	5.1	5.6	94	98	103	15	0.95	0.95	62 500	62 500	4.8	7	9
C-C motif chemokine 15 (CCL15)	Q16663	6.3	7.2	8.2	95	99	95	15	7.63	7.63	31 250	62 500	3.6	9	15
C-C motif chemokine 16 (CCL16)	O15467	5.4	6.2	7.8	97	96	98	15	15.26	15.26	15 625	15 625	3	10	18
C-C motif chemokine 24 (CCL24)	O00175	4.4	5.1	6.4	90	94	105	15	0.95	1.91	7 812	15 625	3.6	9	13
CD166 antigen (ALCAM)	Q13740	5.1	5.3	5.7	94	98	102	15	0.24	0.24	7 812	15 625	4.5	7	8
Chitinase-3-like protein 1 (CHI3L1)	P36222	4.5	5.3	7.3	89	96	107	15	1.91	1.91	3 906	15 625	3.3	8	10
Chitotriosidase-1 (CHIT1)	Q13231	1.8	2.8	5.2	90	93	102	15	15.26	15.26	31 250	62 500	3.3	8	11
Collagen alpha-1(I) chain (COL1A1)	P02452	2.2	2.7	3.4	99	97	98	7.5	244.14	244.14	62 500	62 500	2.4	6	10
Complement component C1q receptor (CD93)	Q9NPY3	9.1	9.7	10.1	98	103	104	15	0.95	1.91	15 625	62 500	3.9	8	11
Contactin-1 (CNTN1)	Q12860	3.4	3.7	4.3	93	95	100	15	3.81	7.63	31 250	62 500	3.6	7	9
C-X-C motif chemokine 16 (CXCL16)	Q9H2A7	6.5	6.8	7.2	93	96	100	15	1.91	3.81	31 250	62 500	3.9	9	12
Cystatin-B (CSTB)	P04080	3.7	4.2	5.4	73	80	102	0.5	0.95	1.91	7 812	15 625	3.6	8	9
Elafin (PI3)	P19957	3	3.5	4.4	93	96	98	15	0.95	15.26	15 625	15 625	3	8	13
Ephrin type-B receptor 4 (EPHB4)	P54760	2.1	2.5	2.7	95	94	100	15	7.63	7.63	62 500	125 000	3.9	8	9
Epidermal growth factor receptor (EGFR)	P00533	2.3	2.6	2.8	98	100	101	15	30.52	30.52	31 250	62 500	3	7	10
Epithelial cell adhesion molecule (Ep-CAM)	P16422	3.3	4.6	6.9	91	98	102	15	0.48	0.95	15 625	62 500	4.2	8	11
E-selectin (SELE)	P16581	11.5	12	13.4	98	99	105	15	0.12	0.12	7 812	15 625	4.8	7	10
Fatty acid-binding protein, adipocyte (FABP4)	P15090	3.6	5.2	6.4	85	89	96	15	1.91	1.91	15 625	62 500	3.9	8	9
Galectin-3 (Gal-3)	P17931	5.2	5.7	6.2	90	95	104	0.9	30.52	30.52	62 500	62 500	3.3	8	9
Galectin-4 (Gal-4)	P56470	3	3.6	4.1	94	90	90	15	3.81	7.63	62 500	62 500	3.9	8	10
Granulins (GRN)	P28799	3.2	3.4	4	93	96	102	15	61.04	61.04	62 500	62 500	3	7	11
Growth/differentiation factor 15 (GDF-15)	Q99988	3.7	4.3	4.9	89	89	91	15	0.95	0.95	15 625	15 625	4.2	9	11
Insulin-like growth factor-binding protein 1 (IGFBP-1)	P08833	2.1	5.2	6.7	96	117	113	15	15.26	15.26	125 000	125 000	3.9	8	10
Insulin-like Growth Factor-Binding Protein 2 (IGFBP-2)	P18065	6.1	7.6	9	97	105	103	15	122.07	122.07	62 500	62 500	2.7	9	15
Insulin-like growth factor-binding protein 7 (IGFBP-7)	Q16270	4	4.3	4.8	85	87	92	15	30.52	61.04	31 250	62 500	2.7	9	13
Integrin beta-2 (ITGB2)	P05107	6.3	6.8	7.4	55	53	59	15	1.91	7.63	62 500	62 500	3.9	8	11
Intercellular adhesion molecule 2 (ICAM-2)	P13598	5	5.5	6	92	101	108	15	30.52	61.04	125 000	125 000	3.3	8	11
Interleukin-1 receptor type 1 (IL-1RT1)	P14778	6.5	7.2	7.4	91	96	100	15	0.03	0.03	3 906	31 250	5.1	8	10
Interleukin-1 receptor type 2 (IL-1RT2)	P27930	5	5.6	6.3	93	99	101	15	0.95	1.91	15 625	62 500	3.9	8	10
Interleukin-17 receptor A (IL-17RA)	Q96F46	3.5	4.6	5.6	89	89	98	15	0.95	0.95	31 250	62 500	4.5	8	10
Interleukin-18-binding protein (IL-18BP)	O95998	6.3	6.7	7.7	93	99	103	15	1.91	1.91	31 250	62 500	4.2	8	10
Interleukin-2 receptor subunit alpha (IL2-RA)	P01589	3.5	3.9	4.5	93	96	101	15	0.06	0.06	1 953	7 812	4.5	8	8
Interleukin-6 receptor subunit alpha (IL-6RA)	P08887	10.4	10.9	11.5	97	99	105	15	0.12	0.24	7 812	15 625	4.5	8	9
Junctional adhesion molecule A (JAM-A)	Q9Y624	4	4.4	5.1	101	95	101	0.5	0.12	0.24	3 906	31 250	4.2	8	11
Kallikrein-6 (KLK6)	Q92876	3	3.6	4.1	89	93	100	15	1.91	1.91	15 625	62 500	3.9	8	11
Low-density lipoprotein receptor (LDL receptor)	P01130	3.7	4.4	5.7	105	89	98	15	1.91	1.91	31 250	31 250	4.2	8	9
Lymphotoxin-beta receptor (LTBR)	P36941	3.4	3.9	4.2	90	94	100	15	0.24	0.48	15 625	15 625	4.5	8	10
Matrix extracellular phosphoglycoprotein (MEPE)	Q9NQ76	3.3	4.2	5.5	65	63	60	15	30.52	30.52	62 500	125 000	3.3	9	21
Matrix metalloproteinase-2 (MMP-2)	P08253	3.8	4.4	5	89	94	94	15	61.04	122.07	62 500	125 000	2.7	9	13
Matrix metalloproteinase-3 (MMP-3)	P08254	6.4	7.5	8.5	108	110	116	15	0.95	0.95	15 625	62 500	4.2	9	14
Matrix metalloproteinase-9 (MMP-9)	P14780	2.7	3.3	4.3	64	116	294	3.8	244.14	244.14	500 000	500 000	3.3	8	12

		Sample types						Endogenous interference	Analytical measurement					Precision	
		Percentile EDTA plasma (NPX)			Relative to EDTA plasma (%)			mg/mL	pg/mL				log10	% CV	
Target	UniProt No	10th	Median	90th	ACD	Heparin	Serum	Haemolysate	LOD	LLOQ	ULOQ	Hook	Range	Intra	Inter
Metalloproteinase inhibitor 4 (TIMP4)	Q99727	4.2	4.9	5.9	88	85	90	15	3.81	7.63	31 250	62 500	3.6	9	12
Monocyte chemotactic protein 1 (MCP-1)	P13500	2.6	3	3.6	100	98	113	15	0.06	0.12	1 953	3 906	4.2	8	12
Myeloblastin (PRTN3)	P24158	4	4.7	5.3	92	150	254	0.9	0.48	7.63	31 250	62 500	3.6	8	14
Myeloperoxidase (MPO)	P05164	4.2	4.7	5.5	99	151	209	3.8	7.63	7.63	7 812	15 625	3	7	8
Myoglobin (MB)	P02144	6	6.7	8.2	92	91	99	15	0.06	0.06	7 812	31 250	5.1	8	15
Neurogenic locus notch homolog protein 3 (Notch 3)	Q9UM47	4.1	4.6	5	91	95	97	15	1.91	3.81	62 500	62 500	4.2	9	10
N-terminal prohormone brain natriuretic peptide (NT-proBNP)	NA	NA	1.5	2.9	82	NA	NA	15	3.81	7.63	15 625	31 250	3.3	11	13
Osteopontin (OPN)	P10451	4.6	5.4	6	82	76	66	15	122.07	122.07	31 250	62 500	2.4	8	11
Osteoprotegerin (OPG)	O00300	3	3.4	4.1	91	94	99	15	0.48	0.95	15 625	31 250	4.2	8	11
Paraoxonase (PON 3) (PON3)	Q15166	5.4	6.5	7.5	71	74	78	15	7.63	7.63	125 000	125 000	4.2	9	13
Peptidoglycan recognition protein 1 (PGLYRP1)	O75594	6.4	6.9	8.1	91	109	175	15	0.95	0.95	15 625	15 625	4.2	8	12
Perlecan (PLC)	P98160	6.2	6.6	7.1	87	91	99	15	7.63	15.26	62 500	125 000	3.6	7	9
Plasminogen activator inhibitor 1 (PAI)	P05121	4.3	5.9	7.2	44	131	239	15	0.95	0.95	15 625	15 625	4.2	8	10
Platelet endothelial cell adhesion molecule (PECAM-1)	P16284	5	5.3	5.7	101	98	103	15	0.95	0.95	15 625	62 500	4.2	7	10
Platelet glycoprotein VI (GP6)	Q9HCN6	NA	0.7	1.1	75	96	203	15	30.52	30.52	31 250	62 500	3	8	18
Platelet-derived growth factor subunit A (PDGF subunit A)	P04085	1.5	2.3	2.9	23	136	298	15	0.95	1.91	15 625	31 250	3.9	9	15
Proprotein convertase subtilisin/kexin type 9 (PCSK9)	Q8NBP7	2.9	3.3	4	104	112	104	15	122.07	122.07	125 000	1 000 000	3	10	25
Protein delta homolog 1 (DLK-1)	P80370	4.3	5.2	6.1	93	101	106	15	3.81	3.81	31 250	62 500	3.9	8	11
P-selectin (SELP)	P16109	8.7	9	9.7	79	104	138	15	0.12	0.48	15 625	15 625	4.5	8	10
Pulmonary surfactant-associated protein D (PSP-D)	P35247	1.8	2.6	4	94	88	90	15	15.26	15.26	62 500	125 000	3.6	9	9
Resistin (RETN)	Q9HD89	4.8	5.8	6.7	93	102	131	15	0.06	0.06	7 812	15 625	5.1	7	13
Retinoic acid receptor responder protein 2 (RARRES2)	Q99969	11	11.8	12.4	94	115	104	15	7.63	7.63	15 625	62 500	3.3	9	11
Scavenger receptor cysteine-rich type 1 protein M130 (CD163)	Q86VB7	7	7.6	8.4	91	97	100	15	3.81	7.63	62 500	62 500	3.9	7	9
Secretoglobin family 3A member 2 (SCGB3A2)	Q96PL1	2.1	3.3	5.3	94	101	103	15	61.04	244.14	500 000	1 000 000	3.3	10	22
Spondin-1 (SPON1)	Q9HCB6	1.7	2	2.7	93	88	92	15	122.07	122.07	62 500	125 000	2.7	8	12
ST2 protein (ST2)	Q01638	3.6	4.5	5.3	93	92	100	15	7.63	7.63	62 500	62 500	3.9	8	11
Tartrate-resistant acid phosphatase type 5 (TR-AP)	P13686	4.8	5.3	5.8	99	96	101	15	1.91	7.63	15 625	62 500	3.3	7	10
Tissue factor pathway inhibitor (TFPI)	P10646	8	8.8	9.3	88	100	91	15	3.81	7.63	31 250	62 500	3.6	9	12
Tissue-type plasminogen activator (t-PA)	P00750	4.4	5	6.2	141	59	123	15	1.91	1.91	62 500	62 500	4.5	9	16
Transferrin receptor protein 1 (TR)	P02786	6.9	7.3	8.2	85	71	71	15	30.52	61.04	125 000	125 000	3.3	6	9
Trefoil factor 3 (TFF3)	Q07654	4.6	5.2	6	92	97	102	15	0.24	0.24	3 906	7 812	4.2	8	9
Trem-like transcript 2 protein (TLT-2)	Q5T2D2	3.9	4.6	5.2	83	96	144	15	3.81	3.81	15 625	31 250	3.6	8	12
Tumor necrosis factor ligand superfamily member 13B (TNFSF13B)	Q9Y275	5.5	6	6.5	90	96	106	15	0.24	0.48	15 625	31 250	4.5	8	12
Tumor necrosis factor receptor 1 (TNF-R1)	P19438	4.8	5.3	5.8	93	98	121	15	3.81	7.63	31 250	62 500	3.6	8	12
Tumor necrosis factor receptor 2 (TNF-R2)	P20333	4.9	5.4	5.9	92	95	104	15	1.91	3.81	31 250	62 500	3.9	8	10
Tumor necrosis factor receptor superfamily member 10C (TNFRSF10C)	O14798	6.1	6.9	7.5	91	96	106	15	0.06	0.06	3 906	7 812	4.8	7	10
Tumor necrosis factor receptor superfamily member 14 (TNFRSF14)	Q92956	4.1	4.6	5.1	92	123	210	15	0.24	0.95	15 625	31 250	4.2	8	10
Tumor necrosis factor receptor superfamily member 6 (FAS)	P25445	4.6	5.1	5.7	95	98	100	3.8	0.48	0.95	15 625	62 500	4.2	8	12
Tyrosine-protein kinase receptor UFO (AXL)	P30530	7.9	8.5	8.9	93	96	97	15	0.48	0.95	15 625	15 625	4.2	8	11
Tyrosine-protein phosphatase non-receptor type substrate 1 (SHPS-1)	P78324	3.6	4	4.5	97	101	103	15	1.91	1.91	15 625	62 500	3.9	8	9
Urokinase plasminogen activator surface receptor (U-PAR)	Q03405	4.2	4.6	5.1	91	101	137	15	0.24	0.24	3 906	62 500	4.2	8	10
Urokinase-type plasminogen activator (uPA)	P00749	4	4.8	5.4	96	98	102	15	0.48	0.95	15 625	31 250	4.2	8	14
von Willebrand factor (vWF)	P04275	5	5.7	7.1	125	133	258	15	0.95	15.26	31 250	62 500	3.3	8	12

2.3 PRECISION

REPEATABILITY

Intra-assay variation (within-run) was calculated as the mean %CV for 6 individual samples run, within each of 10 separate runs during the validation studies. Inter-assay variation (between-run) was calculated between experiments with the same operator. The reported inter-assay mean %CV is the average of three operators' %CV. Variation calculations were performed on linearized values for 92 analytes for which response levels could be measured in serum and normal plasma, see Table 1.

Across all 92 assays, the mean intra-assay and inter-assay variations observed were 8.1% and 11.5%, respectively. The distributions of intra-assay and inter-assay variations are shown in Figure 4.

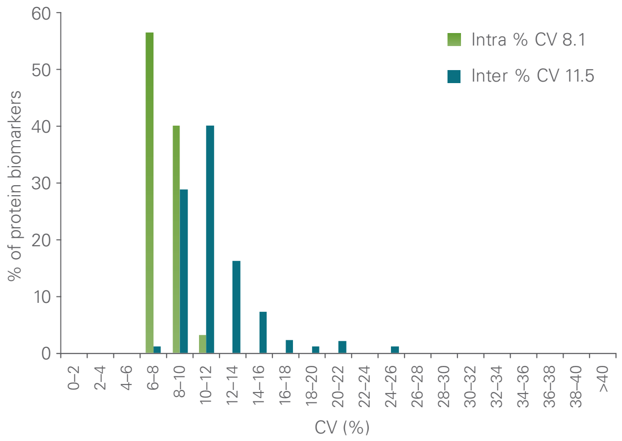

Fig 4. Distribution of intra-assay and inter-assay variations of Olink CARDIOVASCULAR III

REPRODUCIBILITY

Inter-site variations (between-site) were also investigated during the validation in a beta-site study, to estimate expected increase in variation values produced by introducing a 100 fold pre-dilution step of samples prior to running the Olink CVD III assay protocol. Six individual samples were distributed to two laboratories together with Olink CVD III reagent kits. Each site was instructed to perform the analysis of the 6 individual samples according to the same run design and asked to perform two independent runs.

The intra-assay mean CV value results for beta-site 1 and 2 were 5% and 7%, and the mean inter-assay CVs were 16% and 30%, respectively.

Overall, the Olink CVD III showed good reproducibility and repeatability with values in line with Olink results, see Figure 4.

Olink has Analysis Service labs in Sweden and the USA, and in addition there are many Olink-certified core laboratories around the world running the Olink platform (see www.olink.com/service for details). Our experience over several years is that inter-site reproducibility is very good providing that operators are properly trained. For more information please contact support@olink.com.

2.4 ANALYTICAL SPECIFICITY

ASSAY SPECIFICITY

To test the target-protein specificity of the PEA probes used in the panel, all of the antibodies used were tested for cross-reactivity against all of the

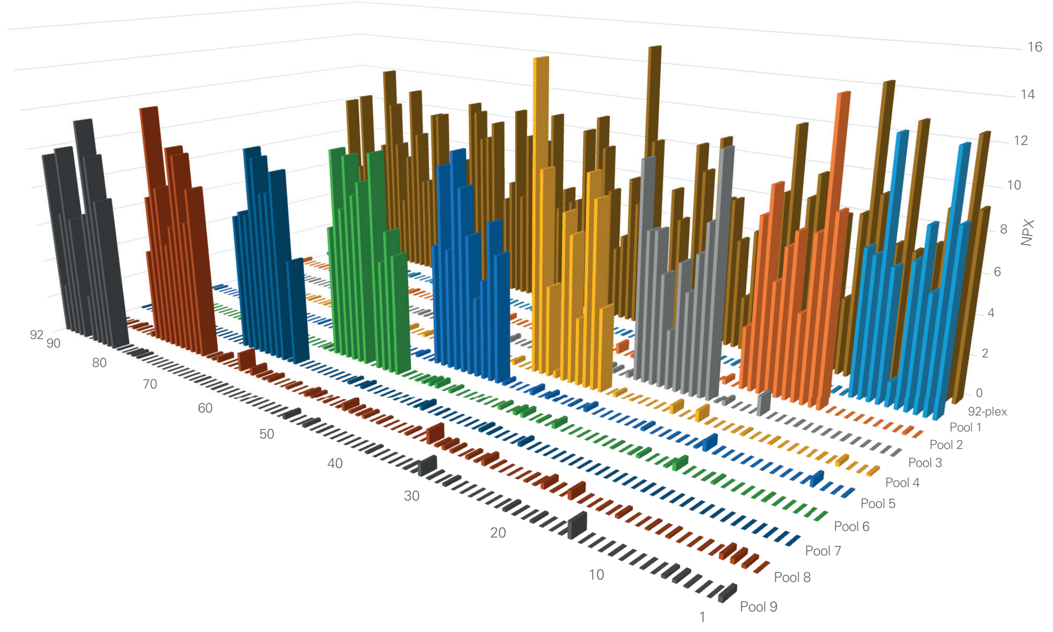

Fig 5. Assay readout specificity of the Olink platform. For each assay, specificity is confirmed by testing antigen sub-pools against the complete 92-plex pool as to each sub-mix.

recombinant proteins used during assay validation. The probes were also checked for cross-reactivity to more than 100 additional proteins (data not shown). This was carried out by creating a test sample consisting of a pool of antigens, which was then incubated with all 92 antibody probe pairs from the panel. To optimize this analysis, 10 sub-pools of antigen were evaluated to cover the 92 assays (see Figure 5).

The lack of significant signal from these tests indicates that each probe pair is specific for its target antigen, demonstrating the readout specificty of the PEA method.

ENDOGENOUS INTERFERENCE

Endogenous interference from heterophilic antibodies, e.g. human anti-mouse antibody (HAMA), and rheumatoid factor are known to cause problems in immunoassays.

To evaluate the potential impact of this specific interference, a special "mismatch" system was designed. The only way to generate a signal in this system is to bring antibody probe pairs into proximity, by cross-binding substances other than antigens, e.g. heterophilic antibodies or rheumatoid factor. No interference due to HAMA or RF could be detected for any of the samples in any of the previously tested panels, indicating sufficient blocking of these agents (data not shown).

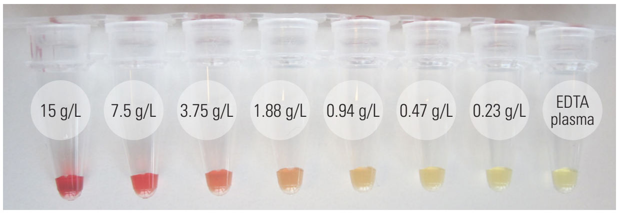

Fig 6. Endogenous interference. Levels tested for hemolysate were 0.23-15 g/L hemoglobin. The highest hemolysate concentration translates to about 10% hemolysis.

The potential impact of some known interfering serum and plasma components was evaluated using serial dilutions of hemolysate, lipids and bilirubin, respectively in EDTA plasma and serum

An example of hemolysate levels tested is shown in Figure 6. These additions simulate different patient health conditions and/or sample collection irregularities. Interference by bilirubin and lipids has previously been evaluated, and disturbance has only been observed at extrem levels corresponding to 8 or 10 times normal [3, 4], values and therefore not performed for Olink CARDIOVASCULAR III. In 22 out of 92 assays, altered values were recorded after the addition of hemolysate. The reason is most likely due to more of the measured analyte leaking out of the disrupted blood cells. A concentration of 15 g/L of hemolysate represents 10% hemolysis of a sample. Table 1 reports the highest concentration of hemolysate that does not have an impact on assay performance.

2.5 SCALABILITY

Assay performance was further evaluated with regard to scalability, meaning the capability of the Olink technology to maintain the same quality of performance irrespective of multiplex grade. Previously, we have shown that a step-wise increase of multiplex grade (8, 24, 48, 72 and 96) does not compromise assay performance (data not shown). To further strengthen that Olink provides consistent results, single assays for Growth Hormone (GH) and Matrix Metalloproteinase (MMP-7) were compared when run in the full Olink CVD II panel. The results for each assay and their observed dCq-values were plotted against the entire 96-plex reaction. The square of the correlation coefficient (R^2) value was generated by linear regression.

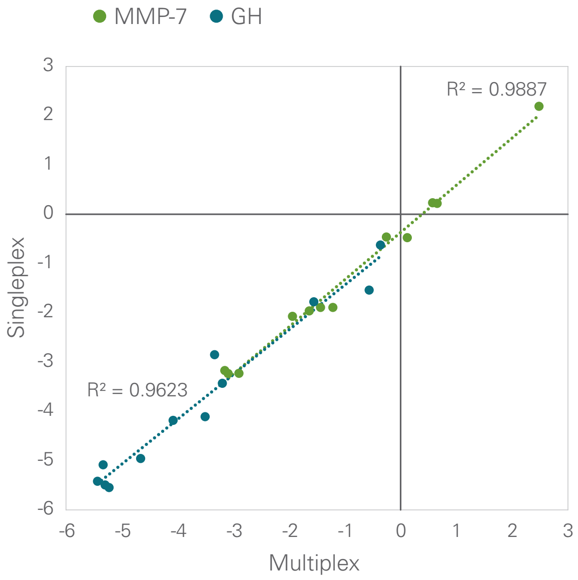

Fig 7. Scalability of the Olink technology platform. This experiment was performed using the Olink CVD II panel. Human plasma samples were analyzed in singleplex for Growth Hormone (GH) and Matrix Metalloproteinase (MMP-7) with the equivalent assays performed in a full 96-plex reaction. The observed dCq (log2) values were plotted, and the correlation coefficient R^2 value was generated by linear regression.

3. References

1. Assarsson E, Lundberg M, Holmquist G, Bjorkesten J, Bucht Thorsen S, Ekman D, Eriksson A, Rennel Dickens E, Ohlsson S, Edfeldt G, Andersson AC, Lindstedt P, Stenvang J, Gullberg M, Fredriksson S. Homogenous 96-Plex PEA Immunoassay Exhibiting High Sensitivity, Specificity, and Excellent Scalability. *PLoS One* April (2014). doi: 10.1371/journal.pone.0095192.

2. Lundberg M, Eriksson A, Tran B, Assarsson E, Fredriksson S. Homogeneous antibody-based proximity extension assays provide sensitive and specific detection of low abundant proteins in human blood. *Nucleic Acid Res* June (2011). doi: 10.1093/nar/gkr424.

3. http://emedicine.medscape.com/article/2074115-overview

4. http://www.nlm.nih.gov/medlineplus/ency/article/003479.htm

TECHNICAL SUPPORT

For technical support, please contact us at support@olink.com

This product is for research use only. Not for use in human diagnostic or therapeutic procedures.

This product includes a license for non-commercial use of Olink products. Commercial users may require additional licenses. Please contact Olink Proteomics AB for details.

There are no warranties, expressed or implied, which extend beyond this description. Olink Proteomics AB is not liable for property damage, personal injury, or economic loss caused by this product.

The following trademarks are owned by Olink AB: Olink® and Olink Bioscience™.

This product is covered by several patents and patent applications including US 6,511,809, US 7,306,904, and related US and foreign patents.

This product is sold under license from PHRI Properties, Inc. and may be used under PHRI Properties patent rights outside the field of human in vitro diagnostics.

Components in the Olink Probe Kit utilise Lightning-Link™ technology and are provided under license from Innova Biosciences.

© 2019 Olink Proteomics AB. All third party trademarks are the property of their respective owners.

1017, v2.1, 2019-12-11

Olink Proteomics
Dag Hammarskjölds v. 52B
SE-752 37 Uppsala, Sweden
www.olink.com
