# Supplementary material for: Plasma proteome profiling of cardiotoxicity in patients with diffuse large B-cell lymphoma
Source: Cardiooncology. 2021 Feb 3;7:6. doi: 10.1186/s40959-021-00092-0 (PMC7856776; doi:10.1186/s40959-021-00092-0)
Supplement: Supplementary file 3 — Additional file 3. [file 40959_2021_92_MOESM3_ESM.pdf]

1. Introduction

Olink® Oncology II is a reagent kit measuring 92 oncology related human protein biomarkers simultaneously. The analytical performance of the product has been carefully validated and the results are presented below.

1.1 TECHNOLOGY

The Olink reagents are based on the Proximity Extension Assay (PEA) technology[1-2], where 92 oligonucleotide labeled antibody probe pairs are allowed to bind to their respective target protein present in the sample. A PCR reporter sequence is formed by a proximity dependent DNA polymerization event, amplified, and subsequently detected and quantified using real-time PCR. The assay is performed in a homogeneous 96-well format without any need for washing steps, see Figure 1.

1.2 QUALITY CONTROLS

Internal and external controls have been developed by Olink for data normalization and quality control purposes. These controls have been designed to enable monitoring of the technical assay performance, as well as the quality of individual samples, providing information at each step of the Olink protocol (see Figure 1). The internal controls are added to each sample and include two Immunoassay controls, one Extension control and one Detection control. The Immunoassay controls (two non-human proteins) monitor all three steps starting with the immunoreaction. The Extension Control (an antibody linked to two matched oligonucleotides for immediate proximity independent of antigen binding) monitors the extension and readout steps and is used for data normalization across samples. Finally, the Detection control (a synthetic double-stranded template) monitors the readout step. Samples for which one or more of the internal control values deviate from a pre-determined range will be flagged and may be removed before statistical analysis.

An external control, inter-plate control (IPC), is included on each plate and used in a second normalization step. This control is made up of a pool of probes similar to the Extension control (Ext Ctrl), but generated with 92 matching oligonucleotide pairs. Furthermore, the improves inter-assay precision and allows for optimal comparison of data derived from multiple runs. The term "Normalized Protein eXpression (NPX)" refers to normalized data as described above.

1.3 DATA ANALYSIS

Data analysis was performed by employing a pre-processing normalization procedure. For each sample and data point, the corresponding Cq-value for the Extension control was substracted, thus normalizing for technical variation within one run. Normalization between runs is then performed for each assay by substracting the corresponding dCq-value for the Interplate Control (IPC) from the dCq-values generated. In the final step of the pre-processing procedure the values are set relative to a correction factor determined by Olink. The generated Normalized Protein eXpression (NPX) unit is on a log2 scale where a larger number represents a higher protein level in the sample, typically with the background level at around zero. Linearization of data is performed by the mathematical operation $2^{\wedge \mathrm{NPX}}$. Coefficient of variation (CV) calculations were performed on linearized values.

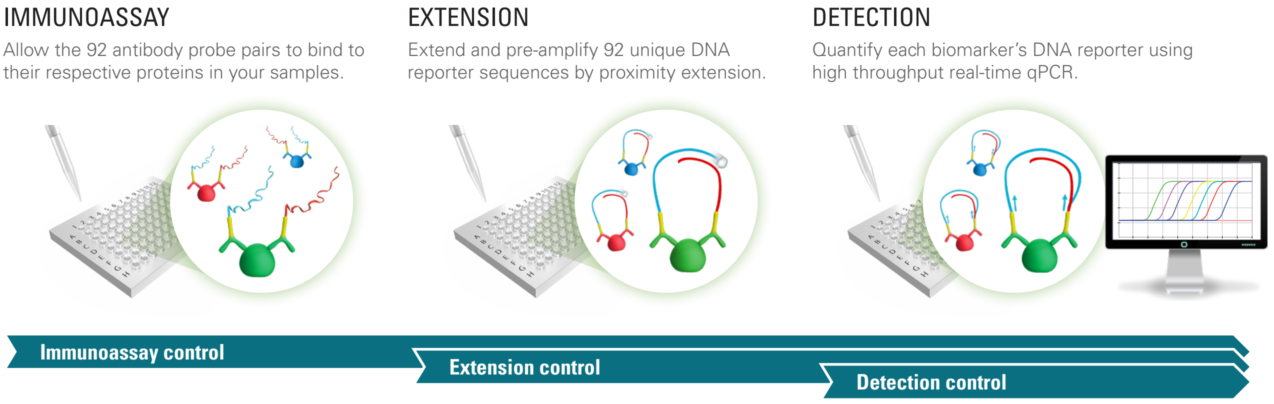

Fig 1. Olink assay procedure (above) and controls (below). The internal controls enables monitoring of the three core steps in the Olink assay and used for quality control and data normalization. Read out is performed by using the Fluidigm® Biomark™ or the Fluidigm® Biomark™ HD system.

2. Performance characteristics

2.1 SAMPLE TYPES

The ability to use different sample types was evaluated with Olink Oncology II by collecting matched serum, EDTA, acid citrate dextrose (ACD), and sodium heparin plasma samples from 4 healthy individuals. Table 1 summarizes response values for 32 normal EDTA plasma samples expressed in NPX, as well as relative differences compared to EDTA plasma. Variations observed between responses in heparin, citrate plasma and serum, as compared to EDTA plasma, were generally small, and all assays will therefore function without limitation in these sample types. In addition, cell lysates from 10 different cell lines were also evaluated.

2.2 ANALYTICAL MEASUREMENT

DETECTION LIMIT

Calibrator curves were determined for 91 out of 92 biomarkers simultaneously in a multiplex format. One protein biomarker (CDKN1A) lacked accessible recombinant antigen. Limit of detection (LOD) was defined as 3 standard deviations above background and reported in pg/mL for all assays where recombinant protein antigen was available, see Table 1 and Figure 2.

HIGH DOSE HOOK EFFECT

The high dose hook effect is a state of antigen excess relative to the reagent antibodies, resulting in falsely lower values. In such cases, a significantly lower value can be reported which leads to misinterpretation of results. Therefore, the hook effect was determined for each analyte, here reported in pg/mL for 91 out of 92 assays, see Table 1.

MEASURING RANGE

The analytical measuring range was defined by the lower limit of quantification (LLOQ) and upper limit of quantification (ULOQ) and reported in order of log10, see Table 1. The upper and lower limits of quantification, ULOQ and LLOQ, respectively were calculated with the following trueness and precision criteria; relative error ≤ 30% and CV ≤ 30%, of back-calculated values, and reported in pg/mL, see Table 1.

Three assays with their analytical data are shown in Figure 2 and the distribution of measuring ranges of 90 assays and endogenous plasma levels are shown in Figure 3. Separate calibrator curves established for each assay may be viewed at www.olink.com/onc2.

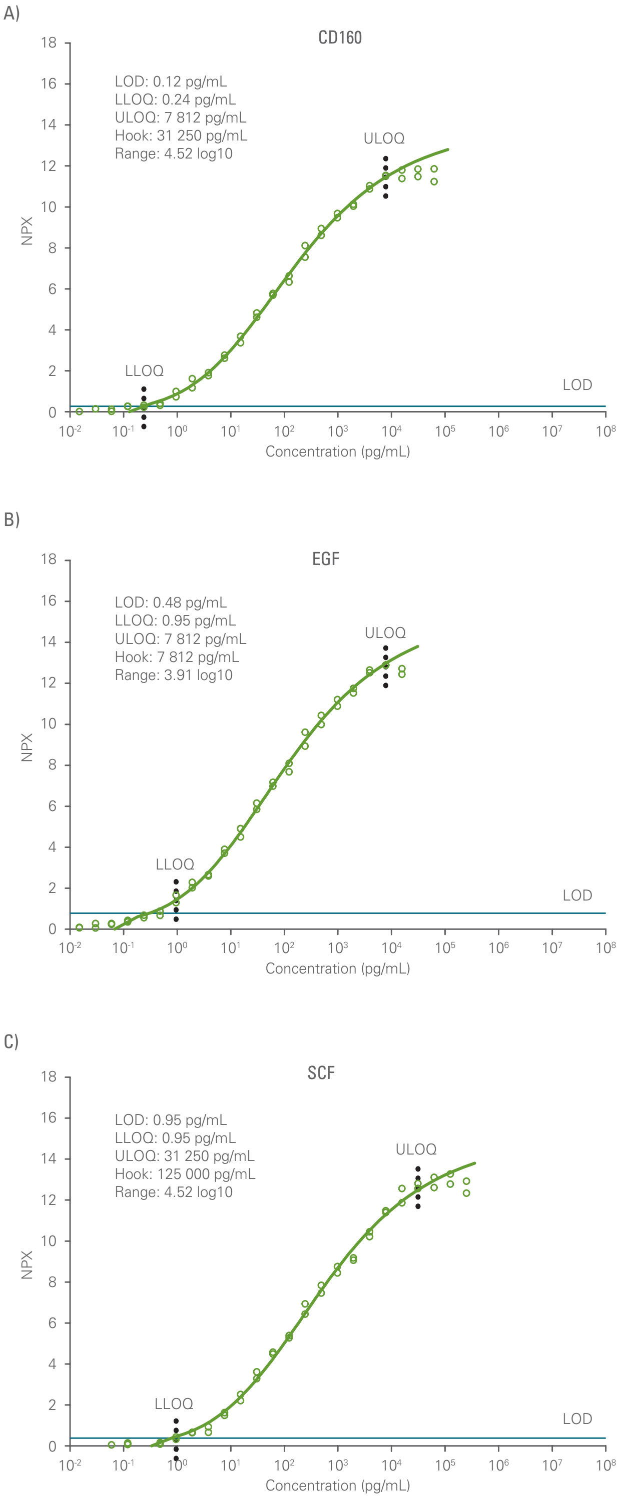

Fig 2. Calibrator curves from 3 assays and their corresponding analytical measurement data.

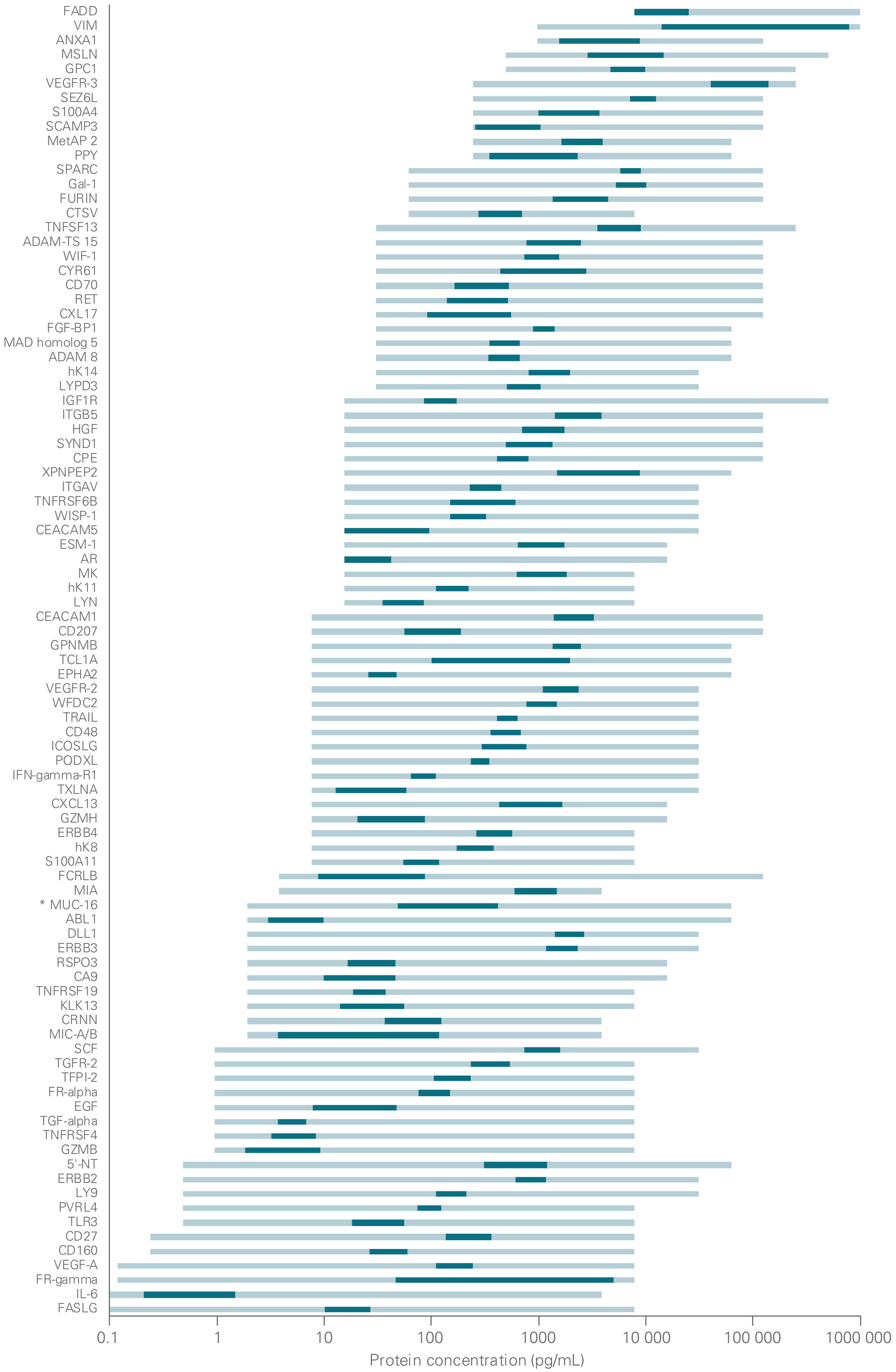

Fig 3. Distribution of analytical measuring range, defined by the lower and upper limits of quantification (LLOQ-ULOQ), and normal plasma levels (dark green bars) for 90 out of 92 analytes. *U/mL.

Table 1. Sample Types; Normalized Protein eXpression (NPX), Endogenous Interference, Analytical Measurement; Limit of Detection (LOD), Lower Limit of Quantification (LLOQ), Upper Limit of Quantification (ULOQ), High Dose Effect (Hook), Range and Precision indicative of assay performance are shown for 92 analytes. Not available, NA

		Sample types						Endogenous Interference	Analytical measurement					Precision	
		Normal plasma levels (NPX)			Relative to EDTA plasma (%)			(mg/mL)	pg/mL				log10	% CV	
Target	UniProt No	10th %tile	Median	90th %tile	ACD	Heparin	Serum	Haemolysate	LOD	LLOQ	ULOQ	Hook	Range	Intra	Inter
5'-nucleotidase (5'-NT)	P21589	8.3	8.9	9.7	105	137	142	7.5	0.5	0.5	62 500	62 500	5.1	6.6	23
A disintegrin and metalloproteinase with thrombospondin motifs 15 (ADAM-TS 15)	Q8TE58	3.1	4.1	4.7	86	56	27	15	15	30	125 000	250 000	3.6	7.6	14
Alpha-taxilin (TXLNA)	P40222	1.5	2.3	3.3	49	32	46	3.8	7.6	7.6	31 250	125 000	3.6	7.3	13
Amphiregulin (AR)	P15514	1.6	2.0	2.9	93	83	121	15	1.9	15	15 625	31 250	3.0	8.8	12
Annexin A1 (ANXA1)	P04083	1.3	2.0	3.5	97	171	232	0.9	976	976	125 000	500 000	2.1	7.8	16
Carbonic anhydrase 9 (CA9)	Q16790	2.1	2.8	3.9	102	101	125	7.5	1.9	1.9	15 625	31 250	3.9	8.2	14
Carboxypeptidase E (CPE)	P16870	3.1	3.6	4.1	87	78	102	15	15	15	125 000	500 000	3.9	8.5	16
Carcinoembryonic antigen-related cell adhesion molecule 1 (CEACAM1)	P13688	6.8	7.4	7.8	95	98	118	15	7.6	7.6	125 000	125 000	4.2	4.9	13
Carcinoembryonic antigen-related cell adhesion molecule 5 (CEACAM5)	P06731	1.7	2.7	3.7	95	88	109	15	7.6	15	31 250	125 000	3.3	11	20
Cathepsin L2 (CTSV)	O60911	3.2	3.7	4.4	96	78	105	15	61	61	7 812	15 625	2.1	8.8	11
CD160 antigen (CD160)	O95971	4.3	5.0	5.6	94	102	118	15	0.1	0.2	7 812	31 250	4.5	7.6	16
CD27 antigen (CD27)	P26842	7.5	7.9	8.6	95	100	109	15	0.1	0.2	7 812	7 812	4.5	7.2	12
CD48 antigen (CD48)	P09326	6.3	6.7	7.2	90	109	119	15	7.6	7.6	31 250	125 000	3.6	6.6	14
CD70 antigen (CD70)	P32970	3.5	4.2	5.0	95	96	165	7.5	15	30	125 000	250 000	3.6	7.2	15
Cornulin (CRNN)	Q9UBG3	4.6	5.6	6.5	99	104	112	15	1.9	1.9	3 906	15 625	3.3	9.4	16
C-type lectin domain family 4 member K (CD207)	Q9UJ71	2.1	2.6	3.4	102	101	111	7.5	7.6	7.6	125 000	250 000	4.2	8.2	14
C-X-C motif chemokine 13 (CXCL13)	O43927	7.1	8.0	9.3	100	82	132	15	7.6	7.6	15 625	15 625	3.3	6.1	11
Cyclin-dependent kinase inhibitor 1 (CDKN1A)	P38936	0.8	1.1	1.9	60	39	44	15	NA	NA	NA	NA	NA	7.3	12
Delta-like protein 1 (DLL1)	O00548	8.5	9.0	9.4	94	103	117	15	1.9	1.9	31 250	125 000	4.2	7.8	15
Disintegrin and metalloproteinase domain-containing protein (ADAM8)	P78325	3.5	4.0	4.4	104	112	153	15	15	30	62 500	125 000	3.3	7.0	16
Endothelial cell-specific molecule 1 (ESM-1)	Q9NQ30	7.0	7.7	8.7	60	53	82	7.5	7.6	15	15 625	31 250	3.0	9.6	14
Ephrin type-A receptor 2 (EPH2)	P29317	1.6	1.9	2.2	98	101	124	15	7.6	7.6	62 500	125 000	3.9	8.0	15
FAS-associated death domain protein (FADD)	Q13158	0.8	1.0	1.7	85	87	128	1.9	7812	7812	1 000 000	1 000 000	2.1	7.2	13
Fc receptor-like B (FCRLB)	Q6BAA4	1.0	1.6	2.8	101	76	103	15	7.8	3.8	125 000	125 000	4.5	8.5	13
Fibroblast growth factor-binding protein 1 (FGF-BP1)	Q14512	6.0	6.2	6.7	116	98	117	15	7.6	30	62 500	125 000	3.3	5.7	13
Folate receptor alpha (FR-alpha)	P15328	6.2	6.6	7.1	97	96	122	7.5	0.5	0.95	7 812	125 000	3.9	8.1	15
Folate receptor gamma (FR-gamma)	P41439	2.6	9.2	12	97	103	130	3.8	0.1	0.11	7 812	125 000	4.8	7.8	13
Furin (FURIN)	P09958	2.6	3.3	4.0	93	96	143	15	61	61	125 000	125 000	3.3	7.4	15
Galectin-1 (Gal-1)	P09382	5.6	6.1	6.5	95	106	118	15	31	61	125 000	250 000	3.3	5.3	14
Glypican-1 (GPC1)	P35052	4.0	4.5	5.1	89	99	121	15	122	488	250 000	500 000	2.7	7.7	13
Granzyme B (GZMB)	P10144	1.4	2.3	3.0	95	73	92	3.8	1.0	0.95	7 812	7 812	3.9	9.1	13
Granzyme H (GZMH)	P20718	2.7	3.6	4.5	99	107	124	1.9	3.8	7.6	15 625	31 250	3.3	7.7	16
Hepatocyte growth factor (HGF)	P14210	5.4	5.8	6.7	72	54	156	15	7.6	15	125 000	125 000	3.9	8.5	15
ICOS ligand (ICOSLG)	O75144	3.8	4.3	4.8	98	137	148	7.5	7.6	7.6	31 250	125 000	3.6	5.7	15
Insulin-like growth factor 1 receptor (IGF1R)	P08069	3.0	3.3	3.8	95	97	144	15	7.6	15	500 000	500 000	4.5	7.9	15
Integrin alpha-V (ITGAV)	P06756	3.4	3.8	4.3	87	88	108	7.5	7.6	15	31 250	125 000	3.3	5.5	13
Integrin beta-5 (ITGB5)	P18084	7.6	8.2	8.8	56	57	68	15	1.9	15	125 000	125 000	3.9	7.3	13
Interferon gamma receptor 1 (IFN-gamma-R1)	P15260	3.2	3.6	3.9	94	102	121	15	3.8	7.6	31 250	125 000	3.6	7.3	15
Interleukin-6 (IL-6)	P05231	1.9	3.0	4.1	99	96	129	15	0.02	0.05	3 906	7 812	4.8	8.3	12
Kallikrein-11 (hK11)	Q9UBX7	4.6	5.1	5.6	93	105	118	15	15	15	7 812	31 250	2.7	7.8	13
Kallikrein-13 (KLK13)	Q9UKR3	2.2	3.3	3.9	116	88	96	15	1.9	1.9	7 812	125 000	3.6	7.3	16
Kallikrein-14 (hK14)	Q9P0G3	5.9	6.8	7.4	95	65	97	15	15	30	31 250	31 250	3.0	9.4	15
Kallikrein-8 (hK8)	O60259	6.0	6.5	7.0	90	106	112	15	3.8	7.6	7 812	125 000	3.0	7.0	14
Ly6/PLAUR domain-containing protein 3 (LYPD3)	O95274	3.7	4.1	4.7	95	104	115	15	15	30	31 250	125 000	3.0	6.3	13
Melanoma-derived growth regulatory protein (MIA)	Q16674	9.4	9.9	10	97	72	104	15	0.95	3.8	3 906	7 812	3.0	7.4	12
Mesothelin (MSLN)	Q13421	1.9	2.7	3.0	100	109	129	15	244	488	500 000	1 000 000	3.0	6.5	20
Methionine aminopeptidase 2 (MetAP2)	P50579	2.42	2.9	4.0	46	16	92	0	244	244	62 500	125 000	2.4	7.2	12
MHC class I polypeptide-related sequence A/B (MIC-A/B)	Q29983, Q29980	1.15	4.1	4.9	101	103	132	15	0.95	1.9	3 906	125 000	3.3	6.4	17
Midkine (Mk)	P21741	5.2	6.0	7.2	49	39	53	15	15	15	7 812	125 000	2.7	9.6	15

		Sample types						Endogenous Interference	Analytical measurement					Precision	
		Normal plasma levels (NPX)			Relative to EDTA plasma (%)			(mg/mL)	pg/mL				log10	% CV	
Target	UniProt No	10th %tile	Median	90th %tile	ACD	Heparin	Serum	Haemolysate	LOD	LLOQ	ULOQ	Hook	Range	Intra	Inter
Mothers against decapentaplegic homolog 5 (MAD homolog 5)	Q99717	3.1	3.8	4.2	100	18	97	15	30	30	62 500	125 000	3.3	7.4	13
Mucin-16 (MUC-16)	Q8WXI7	3.8	4.8	6.7	86	102	127	15	0.03	0.03	1 250	1 250	4.5	7.9	15
Nectin-4 (PVRL4)	Q96NY8	5.6	5.9	6.3	92	97	119	15	0.47	0.47	7 812	7 812	4.2	7.5	15
Pancreatic prohormone (PPY)	P01298	2.0	4.1	5.9	91	91	52	7.5	244	244	62 500	125 000	2.4	9.7	16
Podocalyxin (PODXL)	O00592	3.9	4.3	4.5	97	105	114	7.5	7.6	7.6	31 250	125 000	3.6	4.6	16
Pro-epidermal growth factor (EGF)	P01133	3.7	4.3	6.6	30	140	727	15	0.47	0.95	7 812	7 812	3.9	9.0	11
Protein CYR61 (CYR61)	O00622	3.8	5.0	6.3	170	51	174	15	7.6	30	125 000	500 000	3.6	9.2	14
Protein S100-A11 (S100A11)	P31949	3.0	3.4	4.0	103	108	152	15	3.8	7.6	7 812	125 000	3.0	6.3	14
Protein S100-A4 (S100A4)	P26447	2.5	3.1	3.8	69	96	203	0	122	244	125 000	125 000	2.7	7.0	13
Proto-oncogene tyrosine-protein kinase receptor Ret (RET)	P07949	2.8	3.6	4.4	170	178	228	7.5	7.6	30	125 000	250 000	3.6	7.7	16
Receptor tyrosine-protein kinase erbB-2 (ERBB2)	P04626	6.7	7.1	7.5	91	99	120	15	0.5	0.47	31 250	31 250	4.8	7.4	15
Receptor tyrosine-protein kinase erbB-3 (ERBB3)	P21860	7.6	8.1	8.4	93	88	116	15	0.95	1.9	31 250	125 000	4.2	6.2	16
Receptor tyrosine-protein kinase erbB-4 (ERBB4)	Q15303	4.5	5.0	5.4	97	99	122	15	3.8	7.6	7 812	31 250	3.0	6.3	15
R-spondin-3 (RSPO3)	Q9BXY4	2.4	3.2	3.8	71	71	67	15	1.9	1.9	15 625	31 250	3.9	9.9	13
Secretory carrier-associated membrane protein 3 (SCAMP3)	O14828	1.2	1.6	2.6	87	88	101	0.5	122	244	125 000	500 000	2.7	8.2	15
Seizure 6-like protein (SEZ6L)	Q9BYH1	5.3	5.8	6.2	94	109	127	15	122	244	125 000	250 000	2.7	7.4	15
SPARC (SPARC)	P09486	5.6	5.9	6.	66	113	144	15	30	61	125 000	1 000 000	3.3	5.0	11
Stem cell factor (SCF)	P21583	8.2	8.8	9.4	92	99	113	7.5	0.95	0.95	31 250	125 000	4.5	7.3	13
Syndecan-1 (SYND1)	P18827	5.4	6.1	7.0	90	101	131	15	7.6	15	125 000	125 000	3.9	7.7	14
T-cell leukemia / lymphoma protein 1A (TCL1A)	P56279	3.5	5.2	7.8	54	83	59	3.8	1.9	7.6	62 500	125 000	3.9	9.1	14
TGF-beta receptor type-2 (TGFR-2)	P37173	6.5	7.2	7.6	90	100	121	15	0.95	0.95	7 812	125 000	3.9	8.3	14
Tissue factor pathway inhibitor 2 (TFP1-2)	P48307	7.1	7.7	8.4	73	88	90	15	0.95	0.95	7 812	15 625	3.9	8.8	13
T-lymphocyte surface antigen Ly-9 (LY9)	Q9HBG7	4.9	5.3	5.8	94	106	122	15	0.47	0.47	31 250	31 250	4.8	6.5	14
TNF-related apoptosis-inducing ligand (TRAIL)	P50591	4.5	5.5	6.1	94	99	118	15	0.47	0.47	7 812	125 000	4.2	7.6	15
Toll-like receptor 3 (TLR3)	O15455	1.8	2.0	2.4	98	87	372	3.8	0.95	0.95	7 812	7 812	3.9	7.7	14
Transforming growth factor alpha (TGF-alpha)	P01135	6.3	6.6	7.0	101	103	117	15	7.6	7.6	62 500	125 000	3.9	5.2	16
Transmembrane glycoprotein NMB (GPNMB)	Q14956	7.0	7.3	7.7	98	97	121	15	1.9	7.6	31 250	31 250	3.6	7.9	12
Tumor necrosis factor ligand superfamily member 13 (TNFSF13)	O75888	7.3	7.9	8.5	78	82	101	7.5	7.6	30	250 000	250 000	3.9	7.9	13
Tumor necrosis factor ligand superfamily member 6 (FASLG)	P48023	8.0	8.7	9.4	114	100	120	15	0.03	0.03	7 812	15 625	5.4	8.9	13
Tumor necrosis factor receptor superfamily member 19 (TNFRSF19)	Q9NS68	3.8	4.3	4.8	87	99	137	7.5	0.47	1.9	7 812	7 812	3.6	7.6	15
Tumor necrosis factor receptor superfamily member 4 (TNFRSF4)	P43489	2.4	3.1	3.6	95	99	127	7.5	0.47	0.95	7 812	7 812	3.9	7.5	15
Tumor necrosis factor receptor superfamily member 6B (TNFRSF6B)	O95407	3.4	4.1	5.1	89	70	154	15	7.6	15	31 250	125 000	3.3	8.5	13
Tyrosine-protein kinase ABL1 (ABL1)	P00519	1.6	2.0	2.6	80	60	82	3.8	0.95	1.9	62 500	250 000	4.5	8.2	18
Tyrosine-protein kinase Lyn (LYN)	P07948	1.0	1.3	1.8	84	56	78	3.8	7.6	15	7 812	7 812	2.7	5.6	13
WAP four-disulfide core domain protein 2 (WFDC2)	Q14508	6.8	7.2	7.6	97	106	121	15	7.6	7.6	31 250	125 000	3.6	6.8	13
Vascular endothelial growth factor A (VEGF-A)	P15692	8.6	9.1	9.7	71	88	153	15	0.11	0.11	7 812	15 625	4.8	8.3	13
Vascular endothelial growth factor receptor 2 (VEGFR-2)	P35968	6.2	6.8	7.2	103	94	114	15	7.6	7.6	31 250	125 000	3.6	6.4	19
Vascular endothelial growth factor receptor 3 (VEGFR-3)	P35916	5.8	6.5	6.8	92	101	129	15	122	244	250 000	1 000 000	3.0	6.1	14
VEGF-co regulated chemokine 1 (CXL17)	Q6UXB2	2.9	4.4	5.2	81	79	57	7.5	7.6	30	125 000	250 000	3.6	8.6	20
Vimentin (VIM)	P08670	1.7	2.9	5.1	73	363	644	0.5	976	976	1 000 000	1 000 000	3.0	8.8	15
Wnt inhibitory factor 1 (WIF-1)	Q9Y5W5	4.7	5.3	5.9	87	98	109	15	15	30	125 000	500 000	3.6	9.1	15
WNT1-inducible-signaling pathway protein 1 (WISP-1)	O95388	3.6	4.2	5.0	74	116	212	15	3.8	15	31 250	31 250	3.3	8.2	15
Xaa-Pro aminopeptidase 2 (XPNPEP2)	O43895	6.1	7.4	7.9	100	101	116	7.5	7.6	15	62 500	125 000	3.6	5.6	15

*U/µl

2.3 PRECISION

REPEATABILITY

Intra-assay variation (within-run) was calculated as the mean %CV for 6 individual samples run in triplicates within each of 9 separate runs during the validation studies. Inter-assay variation (between runs) was calculated between experiments with the same operator. The reported inter-assay %CV is the average of three operators' %CV. Variation calculations were performed on linearized values for 92 analytes for which response levels could be measured in serum and normal plasma, see Table 1.

Across all 92 assays, the mean intra-assay and inter-assay variations were observed to be 7.6% and 14.7%, respectively. The distribution of both intra-assay and inter-assay variations are shown in Figure 4.

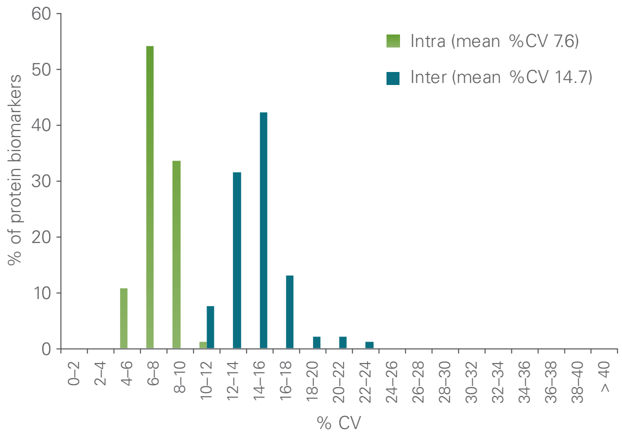

Fig 4. Distribution of intra-assay and inter-assay variations of Olink Oncology II

REPRODUCIBILITY

Inter-site variations (between-site) were investigated during the validation of previous panels in beta-site studies to estimate the expected variations in values between different laboratories, with different operators and using different equipment. The beta-site studies have previously shown reproducibility and repeatability in line with Olink Bioscience results, and were therefore not performed for Olink Oncology II $^{96\times96}$. For information on performed beta-site studies, download our Data Validation documents at www.olink.com/data-validation.

2.4 ANALYTICAL SPECIFICITY

ASSAY SPECIFICITY

To test that the antibodies selected for use in our Olink Oncology II assays are specific for their desired targets, we measured each assay response to all of the 92 panel-specific proteins, as well as against an additional 107 proteins (not shown). In principle, the specificity is tested by creating a test sample, consisting of a pool of antigens, which is then incubated with all 92 antibody probe pairs from the panel. Only if there is a correct match will a reporter sequence be created and serve as a template for subsequent real-time qPCR. Ten sub-pools of antigen are evaluated to cover the 92 assays in Olink, see Figure 5. None of the Olink Oncology II $^{96\times96}$ showed significant signal from the proteins tested.

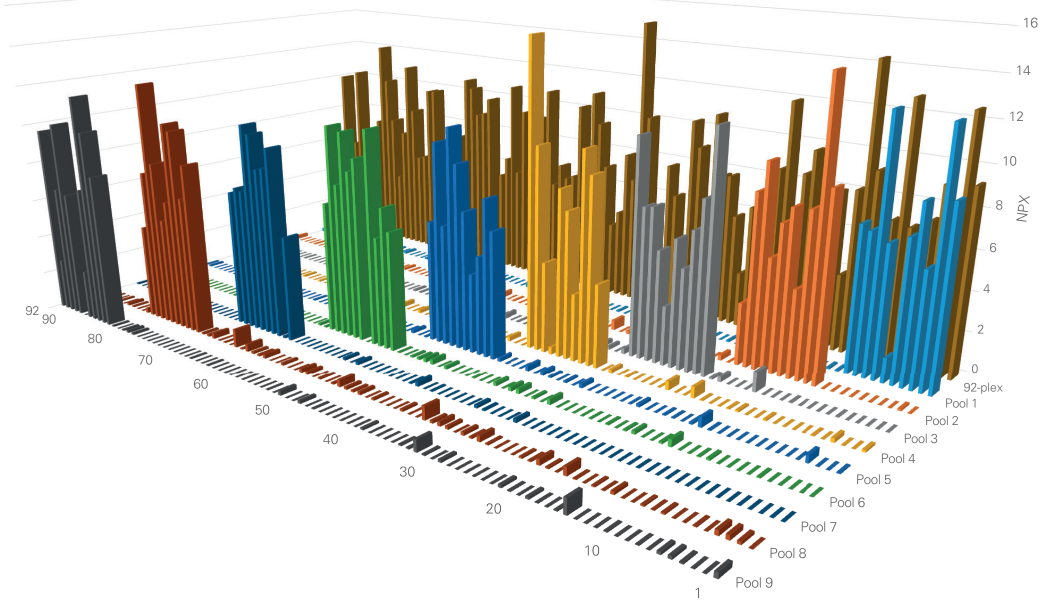

Fig 5. Assay readout specificity of the Olink platform. For each assay, specificity is confirmed by testing antigen sub-pools against the complete 92-plex pool as to each sub-mix.

ENDOGENOUS INTERFERENCE

Endogenous interference from heterophilic antibodies, e.g. human anti-mouse antibody (HAMA), and rheumatoid factor are known to cause problems in some immunoassays. Evaluation of the potential impact of this specific interference has been performed previously using a special "mismatch" system. The only way to generate a signal in this system is by antibody probe pairs being brought into proximity, by cross-binding substances other than antigens, e.g. heterophilic antibodies and similarly acting rheumatoid factor. No interference due to HAMA or RF could be detected for any of the samples in any of the previously tested panels, indicating sufficient blocking of these agents (data not shown).

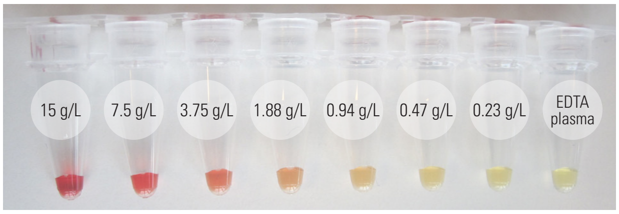

Fig 6. Endogenous interference. Levels tested for hemolysate were 0.23-15 g/L hemoglobin. The highest hemolysate concentration translates to about 10% hemolysis.

The potential impact of bilirubin, lipids and hemolysate, known interfering plasma and serum components, were evaluted at different added concentrations. An example of hemolysate levels tested is shown in Figure 6. These additions represent different patient health conditions and/or sample collection irregularities. Interferens by bilirubin and lipids has previously been evaluated, and disturbance has only been observed at extrem levels corresponding to 8 or 10 times normal [3, 4] values and therefore not performed for Olink Oncology II. In 31 out of 92 assays, altered signal was observed by the addition of hemolysate. The reason is most likely due to actual analyte leaking out of the disrupted blood cells. A concentration of 15 g/L of hemolysate represents 10% hemolysis of a sample. Table 1 reports the highest concentration of hemolysate that does not have an impact on assay performance.

2.5 SCALABILITY

Assay performance was further evaluated with regard to scalability, meaning the capability of the Olink technology to maintain the same quality of performance irrespective of multiplex level. Previously, we have shown that a step-wise increase of multiplex grade (8, 24, 48, 72 and 96) does not compromise assay performance (data not shown). To further strengthen that Olink provides consistent results, single assays for Growth Hormone (GH) and Matrix Metalloproteinase (MMP-7) were compared when run in a full 96-plex reaction. The results for each assay and their observed dCq-values were plotted against the entire 96-plex reaction. The square of the correlation coefficient (R^2) value was generated by linear regression.

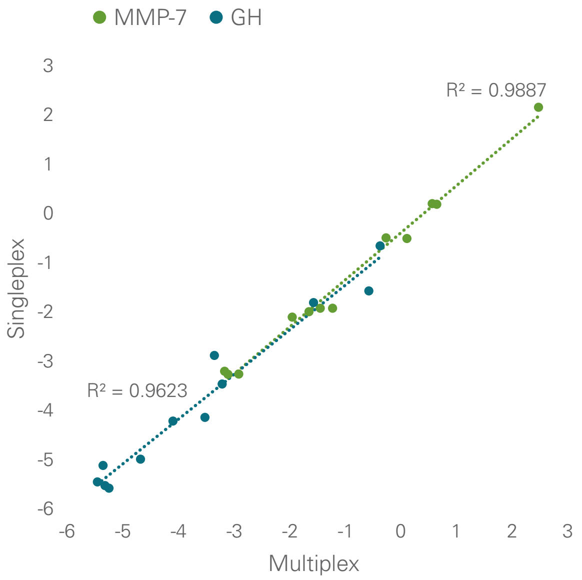

Fig 7. Scalability of the Olink technology platform. The experiment was performed using the Olink CVD II panel. Human plasma samples were analyzed in singleplex for Growth Hormone (GH) and Matrix Metalloproteinase (MMP-7) with the equivalent assays performed in a full 96-plex reaction. The observed dCq (log2) values were plotted, and the correlation coefficient R^2 value was generated by linear regression.

3. References

1. Assarsson E, Lundberg M, Holmquist G, Björkesten J, Bucht Thorsen S, Ekman D, Eriksson A, Rennel Dickens E, Ohlsson S, Edfeldt G, Andersson AC, Lindstedt P, Stenvang J, Gullberg M, Fredriksson S. Homogenous 96-Plex PEA Immunoassay Exhibiting High Sensitivity, Specificity, and Excellent Scalability. *PLoS One* April (2014). doi: 10.1371/journal.pone.0095192.

2. Lundberg M, Eriksson A, Tran B, Assarsson E, Fredriksson S. Homogeneous antibody-based proximity extension assays provide sensitive and specific detection of low abundant proteins in human blood. *Nucleic Acid Res* June (2011). doi: 10.1093/nar/gkr424.

3. http://emedicine.medscape.com/article/2074115-overview

4. http://www.nlm.nih.gov/medlineplus/ency/article/003479.htm

TECHNICAL SUPPORT

For technical support, please contact us at support@olink.com or +46 18 444 3970

For Research Use Only. Not for Use in Diagnostic Procedures.

This product includes a license for non-commercial use of Proseek products. Commercial users may require additional licenses. Please contact Olink Proteomics AB for details.

There are no warranties, expressed or implied, which extend beyond this description. Olink Proteomics AB is not liable for property damage, personal injury, or economic loss caused by this product.

The following trademarks are owned by Olink AB: Olink® and Olink Bioscience™.

This product is covered by several patents and patent applications including US 6,511,809, US 7,306,904 and related US and foreign patents.

This product is sold under license from PHRI Properties, Inc. and may be used under PHRI Properties patent rights outside the field of human in vitro diagnostics.

Components in the Olink Probe Kit utilise Lightning-Link™ technology and are provided under license from Innova Biosciences.

© Copyright 2018 Olink Proteomics AB. All third party trademarks are the property of their respective owners.

1048, v2.0, 2018-02-14

Olink Proteomics
Dag Hammarskjölds v. 52B
SE-752 37 Uppsala, Sweden
www.olink.com
